# Role of Water in Modulating the Fe^3+^/Fe^2+^ Redox Couple in Iron-Based Complexes and Single-Atom Catalysts

**DOI:** 10.1021/acs.jpclett.5c02424

**Published:** 2025-09-18

**Authors:** Alessandro Bonardi, Shuai Xu, Giovanni Di Liberto, Gianfranco Pacchioni

**Affiliations:** † Department of Materials Science, 9305 University of Milano-Bicocca, Via Cozzi 55, 20125 Milano, Italy; ‡ School of Water and Environment, 66350 Chang’an University, 710064 Xi’an, P.R. China

## Abstract

A key challenge in modeling electrocatalysis with single-atom catalysts (SACs) is accurately capturing the redox behavior of transition metals across oxidation states. This is particularly true for iron, a widely used element in such systems. Iron phthalocyanine (FePc) serves as a model compound for graphene-based Fe SACs and is commonly used in reactions like the oxygen evolution reaction (OER). While FePc initially contains Fe­(II), the active species under oxidative conditions is Fe­(III), with an Fe­(II)/Fe­(III) transition occurring at intermediate potentials. Density functional theory (DFT) simulations must reflect this redox change. However, standard DFT predicts that oxidation removes an electron from the ligand, leaving the iron in the II state. This limitation arises not from DFT itself, but from an incomplete model. We show that adding at least one (preferably two) water molecules to the axial coordination sites of iron corrects this issue. The water ligands raise the energy of iron orbitals, making electron removal from the metal more favorable. This finding has two key implications: (1) the redox properties of transition metal complexes and graphene-based SACs are strongly influenced by the coordination environment, including solvent molecules; and (2) accurate description of the atomistic structure of the catalyst requires the explicit inclusion of axial water ligands, not just the in-plane ligands, to capture the true redox behavior.

## Graphical abstract

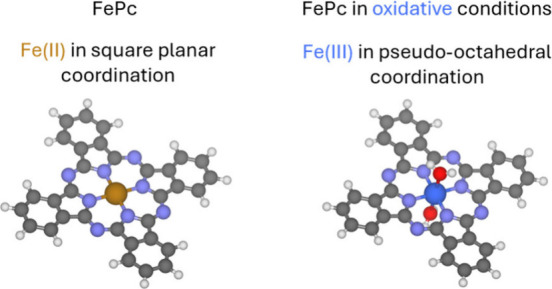

Catalysis plays a major role in the chemical industry. More than 90% of chemical processes of industrial interest involve the use of a catalyst, which is usually in solid phase.^1^ Most of these processes are performed in harsh conditions.^2–4^ Electrocatalysis offers the possibility to carry out reactions in ambient conditions and to promote highly endergonic chemical processes.^5,6^ There are several key processes in this respect. To name a few, one can mention the evolution of hydrogen and oxygen from water,^7–12^ the CO_2_ reduction to fuels,^13–17^ nitrate and N_2_ reduction to ammonia,^18,19^ and electrochemical synthesis of organic compounds.^20,21^ The typical catalysts are based on precious and critical metals, raising some questions about the costs and the sustainability of the processes.

Single-atom catalysis is emerging as a potential solution to this problem.^22–24^ A single-atom catalyst (SAC) consists of metal atoms (usually transition metals, TM) dispersed atomically on a given support.^25–33^ Among others, carbon-based materials, such as nitrogen-doped graphene (N-Gr) and carbon nitride (CN), are widely adopted to stabilize the active species.^34–37^ The metal atom is embedded in the matrix via chemical bonds, leading to specific coordination environments that affect the catalytic activity.^38–41^ A common coordination scheme is represented by a metal atom coordinated by four nitrogen atoms (M-N_4_).^42^ This is well documented for nitrogen-doped graphene, and is emerging for CN as well.^43–45^

In many of the reactions where SACs are involved, the process is based on electron exchange with oxidation or reaction of the TM. The redox properties of the TM-based SACs are essential for the catalytic activity.

In this context iron-based carbon SACs are particularly studied because of a series of advantages, as discussed in some relevant reviews and articles published recently.^25,46,47^ For instance, Wang et al. demonstrated that atomically dispersed iron in CN (Fe@CN) can lower the energy barrier for Li_2_S delithiation, accelerating the electrochemical conversion kinetics in Li–S batteries. This enhanced activity enables faster charge/discharge rates and long-term cycling stability, highlighting the potential of Fe-based single-atom catalysts also for energy storage applications.^48^

Notably, SACs based on graphene or CN supports have a lot in common with coordination chemistry compounds, bridging the two classical worlds of homogeneous and heterogeneous catalysis.^49–54^ Previous studies have shown that the free energy of adsorption of various species on phthalocyanine or porphyrin TM complexes exhibits a linear relationship with the adsorption energies on pyrrolic or pyridinic N-doped graphene.^55^ This correlation supports the notion that the fundamental electronic properties of the TM centers are preserved across both classes of systems. Metal phthalocyanines (Pc) and porphyrins (Pp) are widely used in electrocatalysis essentially for the same reasons.

Fe embedded in a tetraphenylporphyrin is an efficient catalyst for the electrochemical reduction of CO_2_ to CO, especially once OH groups are introduced on the phenyl substituents.^56^ Similarly, iron phthalocyanines (FePc) are active for the reduction of CO_2_, NO_3_^–^ and for the O_2_ evolution.^57–60^ The oxygen evolution reaction is the anodic part of several key reduction processes, including the water splitting reaction.^61–63^ Fe-SACs and FePc are both widely used for this purpose, showing that the key part of the process is related to the Fe local coordination.^25,64–66^ As we mentioned above, Ye et al. provided recently compelling theoretical and experimental evidence of the validity of using Metal phthalocyanines as model systems for heterogeneous metal SACs.^55^

Not surprisingly, the redox properties of Fe SACs or FePc complexes have attracted a lot of interest from the computational chemistry community. Needless to say, a proper description of the redox potential (*V*) of the Fe^2+^/Fe^3+^ couple is of fundamental importance for the prediction of the activity of Fe-based catalysts in electrochemical processes. However, achieving this is more challenging than it might initially seem. In FePc, the iron atom is in II oxidation state (Fe^2+^) and has a square planar coordination with four nitrogen atoms.^67^ Under oxidation conditions, however, for instance at the OER equilibrium potential, the active phase consists of Fe in III oxidation state (Fe^3+^); an Fe^2+^/Fe^3+^ transition occurs at *V* ∼ 0.6–0.8 V (vs Standard Hydrogen Electrode SHE).^68–71^ As we will demonstrate below, this seemingly straightforward process is not accurately captured by standard Density Functional Theory (DFT) calculations. Specifically, in the case of the FePc complex, the removal of an electron leads to oxidation primarily within the ligand sphere, while the Fe center remains in the II oxidation state.^67,72^ This outcome contradicts experimental observations and can potentially lead to incorrect conclusions regarding reaction mechanisms and the energetics of the process.

In this study, we demonstrate that the failure of standard DFT to correctly describe the redox behavior of Fe-based systems is not primarily due to the approximate nature of the exchange-correlation functional. Instead, the root cause of the problem lies in the physical model itself, specifically, the actual coordination environment of the Fe atom in carbon-based SACs and Fe-based complexes. We show that water plays a critical role in determining the redox properties of the Fe^2+^/Fe^3+^ couple by acting as a ligand that can alter the relative stability of Fe 3d orbitals with respect to ligand-based electronic levels.

In order to corroborate this, we have modeled an FePc complex with explicit water molecules, as microsolvation not only offers a practical way to account for explicit solvent effects with acceptable computational costs, it also provided better agreement with experiments than implicit solvation schemes.^73–75^ We found that the inclusion of just one or two water molecules, shifting the Fe coordination geometry from square planar to square pyramidal or octahedral, is sufficient to recover the correct oxidation behavior and quantitatively reproduce the Fe^2+^/Fe^3+^ redox potential.

This leads to a key conclusion: the active species in the reaction is not the four-coordinate Fe center in the bare FePc complex, but rather the five- or six-coordinated Fe in FePc­(H_2_O)_1–2_. Consequently, accurate quantum chemical modeling of FePc chemistry must explicitly account for the presence of water, a conclusion that holds true also for Fe@N-Gr and Fe@CN SACs as well and highlights the crucial role of ligands in defining the metal center state, as already shown in previous studies which investigated key aspects in other metal phtalocyanines single-site catalysts.^76,77^

*Computational Details*. We performed spin-polarized density functional theory calculations, as implemented in the Gaussian package.^78^ We performed calculations using two different exchange and correlation functionals to check for the consistency of the conclusions. We adopted the PBE0 model,^79,80^ one of the most robust DFT functionals,^81^ and the B3LYP,^82,83^ which has been a choice of election for molecular systems over the last three decades. Dispersion interactions have been included by means of Grimme’s D3 scheme.^84^ This computational setup implies an error bar of about 0.1–0.2 eV with respect to benchmark calculations at higher levels of theory.^85–87^ All atoms in the systems were treated with the 6–31G­(d,p)^88–91^ basis set, a double-ζ Pople-type basis augmented by polarization functions on both hydrogens and heavy atoms. The Computational Hydrogen Electrode (CHE) approach was used to predict free energies,^92–94^ by including zero-point energy and entropic correction terms. Zero-point energies were calculated withing the harmonic approximation and entropies were obtained through the formalism of partition function. We report the calculated working quantities, as well as the Cartesian coordinates of the simulated models, in the ESI.

In iron-phthalocyanine, Fe donates two electrons to the ligand, that assumes a charge −2, forming an Fe­(II) species; the system has a triplet ground state, with two unpaired electrons localized in the 3d shell of the Fe atom.^95–98^ The Fe atom has a 4s^0^3d^6^ configuration and the square planar ligand field removes the degeneracy of the 3d orbitals giving rise to a (3d_*xy*_)^2^(3d_z2_)^2^(3d_*xz*_)^1^(3d_*yz*_)^1^(3d_x2‑y2_)^0^ configuration.^99,100^ A way to check the charge state of iron in the complex is to look at the number of unpaired electrons. In fact, while Fe­(II) has a triplet ground state, Fe­(III) gives rise to a quartet and the configuration becomes (3d_z2_)^2^(3d_*xy*_)^1^(3d_*xz*_)^1^(3d_*yz*_)^1^(3d_x2‑y2_)^0^.^95–98^ Thus, the presence of 2 or 3 unpaired electrons is a fingerprint of the Fe^2+^ or Fe^3+^ nature of iron in the system.^95–98^ This specification is necessary since atomic charges, such as those arising from the Quantum Theory of Atoms In Molecules (QTAIM), provide only a rough estimate due to the well-known problem of electron counting in chemical systems.^101^ The configuration of Fe­(II) can also be classified as d^6^L, where the 3d occupation is specified and L represents the ligand shell; according to this nomenclature, Fe­(III) in FePc is d^5^L. This is required to specify when the removal of one electron from the neutral system involves the Fe atom or the ligand L.

In the FePc complex the highest occupied molecular orbital (HOMO) is mainly localized on the ligand, see Figure 1a, irrespective of the level of theory adopted.^102^ Not surprisingly, if we remove one electron from the system, this involves the HOMO and the most stable configuration is a d^6^ L^–1^ quartet, where iron remains in a II oxidation state (Fe^2+^). Thus, the oxidation of FePc does not lead to Fe^3+^, since it is the ligand that get oxidized. This contrasts with the experimental evidence. Another possible solution is the doublet state, very close in energy (Δ*E* = 0.28 eV), but also in this case iron remains Fe­(II). The Fe­(III) state, d^5^L, is significantly higher in energy being more than one eV above the ground state (Δ*E* = 1.30 eV), Table 1. According to this result, the oxidation of FePc does not correspond to the Fe^2+^ to Fe^3+^ transition. This does not depend on the choice of the exchange-correlation functional: similar results are obtained using the B3LYP functional, with deviations of at most 0.3 eV, see Table S1.

**Figure 1:**
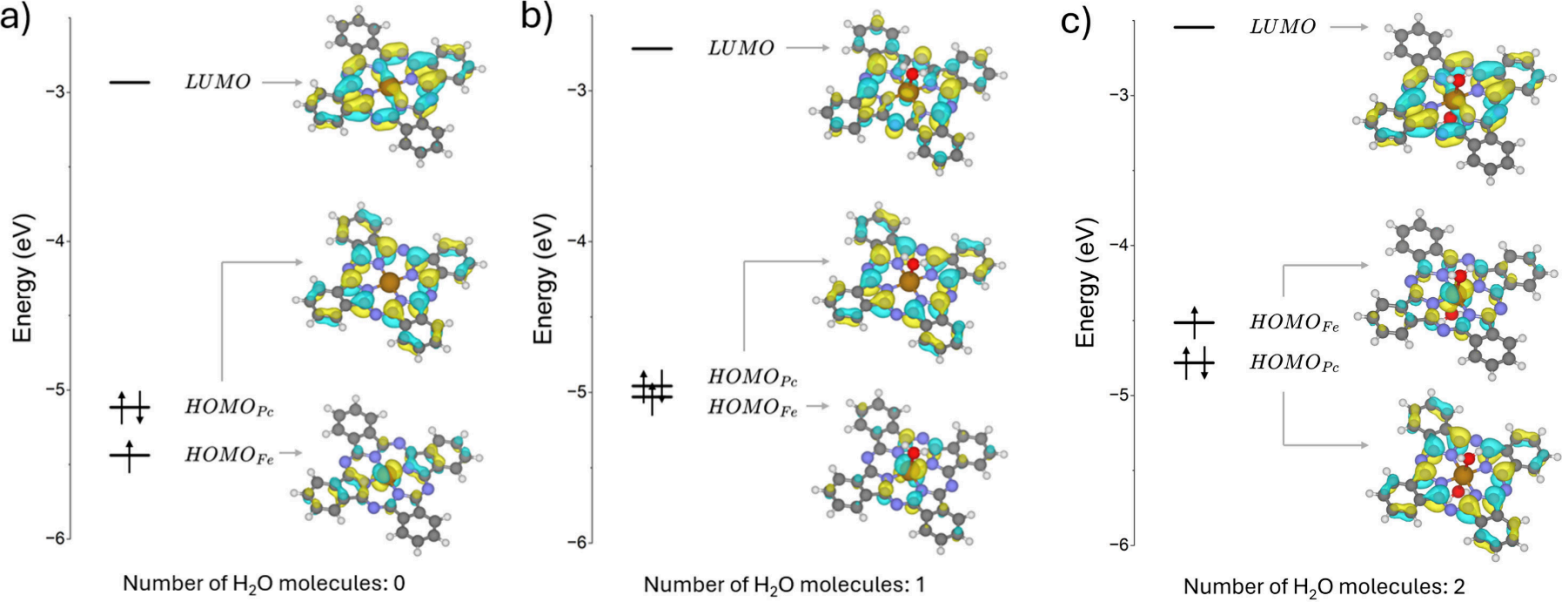
Orbitals energy diagram and [FePc] (a), [FePc­(H_2_O)_1_] (b), and [FePc­(H_2_O)_2_] (c) at PBE0 level.

**Table 1:** Calculated Properties of FePc, FePc­(H_2_O)_1_, and FePc­(H_2_O)_2_ Complexes in Oxidative Conditions^a^

Charge state		0		+1		+2	
		**Triplet**	Quintet	**Quartet**	Sextet	**Triplet**	Quintet
FePc(H_2_O)_0_	Δ*E*	**0**	0.84	**0**	1.3	**0**	0.04
	_μB_	**2.2**	2.0	**2.0**	3.0	**2.0**	3.0
	Config.	d^6^L	d^6^L	d^6^L^–1^	d^5^L	d^6^L^–2^	d^5^L^–1^
		**Triplet**	Quintet	**Quartet**	Sextet	Triplet	**Quintet**
FePc(H_2_O)_1_	Δ*E*	**0**	0.31	**0**	0.94	0.52	**0**
	_μB_	**2.2**	3.9	**3.0**	3.0	2.2	**3.0**
	Config.	d^6^L	d^6^L	d^5^L	d^5^L	d^6^L^–2^	d^5^L^–1^
		**Triplet**	Quintet	Doublet	**Quartet**	Triplet	**Quintet**
FePc(H_2_O)_2_	Δ*E*	**0**	0.68	0.72	**0**	0.72	**0**
	_μB_	**2.3**	3.0	1.0	**3.0**	1.0	**3.0**
	Config.	d^6^L	d^5^L^+1^	d^5^L	d^5^L	d^5^L^–1^	d^5^L^–1^

a Δ*E* are relative energies (in eV) with respect to the most stable state. μ_B_ is the magnetization of the Fe atom. Values are obtained at PBE0 level. The ground state is in bold.

If one further oxidizes the system, thus removing a second electron, (FePc^2+^), then the formation of Fe^3+^ becomes possible, as the two states, d^6^L^–2^, and d^5^L^–1^, are close in energy. Actually, in the most stable state the electron is still preferentially removed from the ligands, d^6^L^–2^, but the d^5^L^–1^ (Fe^3+^) configuration is only 0.04 eV (PBE0) or 0.20 eV (B3LYP) higher in energy. Notice that this result is perfectly in line with other studies. For instance, Liao et al. demonstrated that the HOMO of FePc belongs to the ligands, and thus the oxidation of the system is not expected to occur on the metal.^67^

Of course, square-planar TM complexes as well as heterogeneous SACs consisting of TM embedded in graphene-based materials have similar local structures and can bind and coordinate other molecules in the remaining positions above and below the ligand plane. When this occurs, the coordination becomes square-pyramidal or octahedral-like. Therefore, we first considered a system where the FePc complex coordinates a single water molecule in one of the two possible axial sites, orthogonal to the molecular plane, Figure 1b. In the ground state of the system, FePc­(H_2_O)_1_, Fe is still Fe^2+^ and the ground state is a triplet, Table 1. Things change completely when one removes one electron from this complex. In fact, the resulting most stable configuration is a quartet where the electron is now removed from the Fe atom, leading to a Fe^3+^ state (d^5^L). The presence of one water molecule coordinated to FePc is sufficient to change the picture and to make it compatible with the experimentally observed behavior.

To better understand the origin of the result we analyzed the molecular orbitals of FePc (Figure 1a) and FePc­(H_2_O)_1_ (Figure 1b). Water destabilizes and raises the energy of the occupied frontier molecular orbital mainly consisting of Fe 3d levels, making it nearly isoenergetic with the corresponding one of the L ligand. In FePc the frontier orbitals with dominant Fe character are 0.30 eV (PBE0) and 0.20 eV (B3LYP), respectively, below those of the Pc ligand. In FePc­(H_2_O)_1_, the same orbitals are nearly degenerate (0.03 eV energy difference at both PBE0 and B3LYP levels). This explains the origin of the favorable removal of one electron from the Fe center thereby leading to Fe­(III).

If we consider the presence of two water molecules in a pseudo-octahedral coordination shell, the situation is similar. In this configuration the HOMO of the system is dominated by Fe 3d states and is located 0.27 eV above the Pc levels, Figure 1c. Not surprisingly, going from FePc to FePc­(H_2_O)_2_ model systems, the correct physical picture is recovered and the oxidation of the neutral complex occurs removing one electron from the Fe center, and not from the ligand, Table 1. It must be mentioned that the local coordination is then largely governed by the solvent, but it is also influenced by the nature of the support, as if solvent permeation cannot be assumed, a square-pyramidal geometry would be the only feasible one and the role of the support itself should be explicitly taken into account. In this context, the possible intercalation of water molecules between the metal center and the support would also deserve further investigation.

Once the result has been rationalized qualitatively, we attempt to provide a quantitative estimate of the redox properties of the Fe­(II)/Fe­(III) couple in FePc complexes. Notice that the same arguments apply also to the broad field of SACs consisting of Fe atoms embedded in nitrogen-doped graphene or carbon nitride. We estimated the oxidation potential of the Fe^2+^/Fe^3+^ pair against SHE. We first calculated the free energy to remove one electron from the system with respect to the vacuum level, by including entropic and zero-point energy contributions and correcting the value with the absolute value of the SHE against the vacuum level. This term is calculated by fully relaxing the atomic coordinates of the systems and of the water clusters. Therefore, it explicitly considers the energetic term related to the change in oxidation state of the metal atoms and to some extent the reorganization energy of the water clusters.

$$V_{{\mathrm{Fe}}^{2+}/{\mathrm{Fe}}^{3+}}=\Delta E_{\mathrm{DFT}}-T\Delta S+\Delta E_{\mathrm{ZPE}}=\left(E_{{\left[\mathrm{FePc}\right]}^{+}}-E_{\left[\mathrm{FePc}\right]}\right)-T\left(S_{{\left[FePc\right]}^{+}}-S_{\left[\mathrm{FePc}\right]}\right)+\left({\mathrm{ZPE}}_{{\left[\mathrm{FePc}\right]}^{+}}-{\mathrm{ZPE}}_{\left[\mathrm{FePc}\right]}\right)$$

*E*_[FePc]_ and *E*_[FePc]+_ are the DFT energies of the FePc systems, TS_[FePc]_ and TS_[FePc]+_ are the entropic corrections and ZPE_[FePc]_ and ZPE_[FePc]+_ are the corresponding zero-point energies of the complexes. Finally, the main approximation of the calculation is the neglection of the contribution of bulk water, which is the main reason why we performed a systematic assessment of the calculated potential on the size of the water clusters.

With this approach, the calculated *V* for the “dry” FePc complex is ∼1.2 V at both PBE0 and B3LYP levels. This value considerably overestimates the experimental one, *V* ∼ 0.6–0.8 V.^68–71^ Any attempt to study reaction profiles that involve the Fe/II)/Fe­(III) transition is going to be affected by this error. Next, we considered FePc coordinated to an increasing number of water molecules. We have shown above that it is sufficient to add one water molecule to the complex to change the physical nature of the ionized state, and to get the correct Fe^2+^/Fe^3+^ transition. However, the potential required for this redox process using the FePc­(H_2_O)_1_ model is still around 1.2 eV, much larger than in the experiment. It is only when also the second water molecule is added, FePc­(H_2_O)_2_, and the Fe atom becomes coordinatively saturated, that the Fe^2+^/Fe^3+^ redox potential decreases to *V* = 0.7 V, Table 2, very close to the experimental value, *V*∼0.6–0.8 V.

**Table 2:** Calculated Oxidation Potential, *V* vs SHE of the Fe^2+^/Fe^3+^ Couple in Pc (PBE0/6-31G­(d,p)+D3)

System	*V*/V
[FePc]/[FePc]^+^	1.2
[FePc(H_2_O)]/[FePc(H_2_O)]^+^	1.2
[FePc(H_2_O)_2_]/[FePc(H_2_O)_2_]^+^	0.7
[FePc(H_2_O)_4_]/[FePc(H_2_O)_4_]^+^	0.7
[FePc(H_2_O)_6_]/[FePc(H_2_O)_6_]^+^	0.5
[FePc(H_2_O)_8_]/[FePc(H_2_O)_8_]^+^	0.5
[FePc(H_2_O)_10_]/[FePc(H_2_O)_10_]^+^	0.5
[FePc(H_2_O)_12_]/[FePc(H_2_O)_12_]^+^	0.6
Exp.	**0.6–0.8**

This underlines the critical role of water has in modulating the chemistry of this kind of complexes. Water acts not merely as solvent, but as a ligand capable of locally modifying the electronic structure of the metal center. Similar arguments apply to carbon-based SACs.

The interaction of water with metal complexes under electrochemical conditions is inherently complex, due to the dynamic nature of these interactions and the variety of possible water coordination modes.^103–105^ While a full treatment of this complexity would require extensive ab initio molecular dynamics simulations,^13,106–109^ which is beyond the scope of this work, we nonetheless evaluated the stability of the computed redox potential for the FePc­(H_2_O)_*x*_ model upon the addition of up to 12 water molecules.

Water molecules rapidly form a solvation shell, and the calculated redox potential stabilizes around 0.5–0.6 V (Figure 2, Table 2), which agrees reasonably well with the experimental value, considering the inherent limitations of the DFT method. However, the potential also exhibits notable fluctuations, up to 0.3 eV, depending on the number of water molecules included. These results highlight the need for a more accurate representation of catalyst–solvent interactions to quantitatively reproduce key properties of transition metal complexes such as their redox potential. Nevertheless, a semiquantitative understanding can be achieved by simply adding two water molecules to the vacant coordination sites.

**Figure 2:**
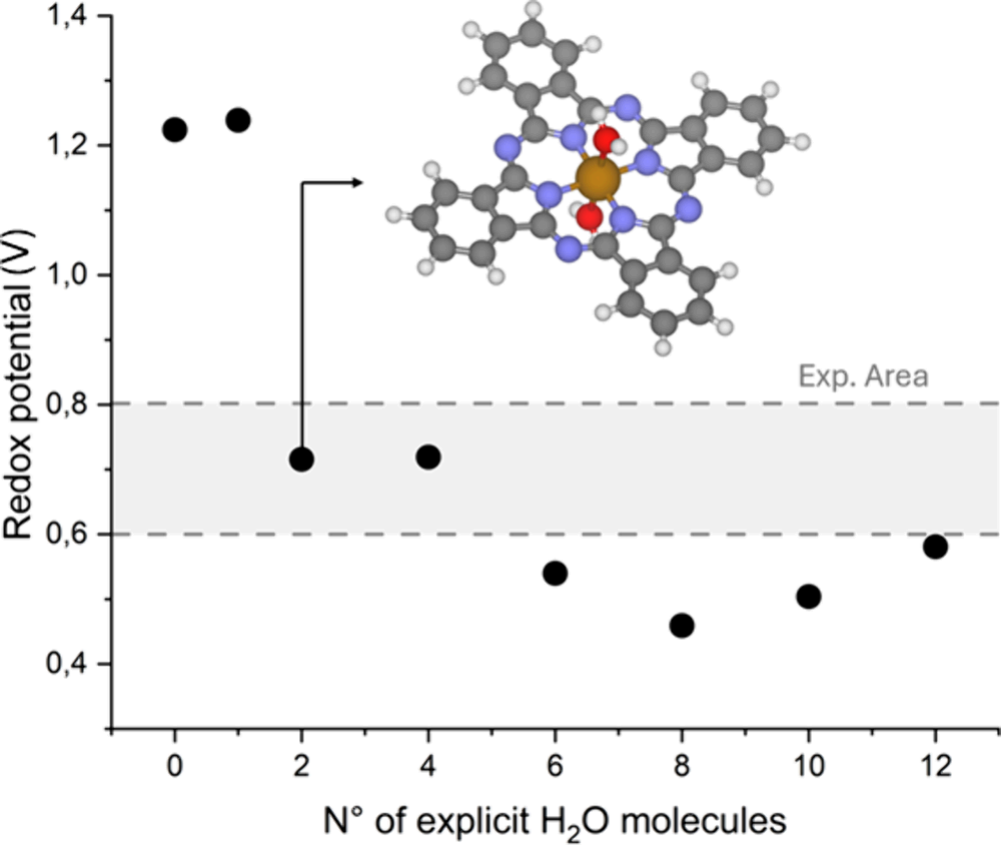
Redox potential (V) for the FePc­(H_2_O)_*x*_ complexes depending on the number of explicit water molecules added as microsolvation shell. The dotted lines indicate the experimental range.

An important aspect to assess is the formation of *OH species resulting from the oxidation of adsorbed water at the oxidation potential of FePc. We calculated the formation energy of *OH starting from two models having one and two adsorbed water molecules, respectively, on [Fe­(III)­Pc]^+^; this corresponds to a square pyramidal and an octahedral-like coordination, respectively. The formation of *OH is endergonic at *V* = 0.80 V, although the value depends on the specific model adopted. The calculated Gibbs free energies are 0.28 and 0.75 eV for [Fe­(III)­Pc­(H_2_O)]^+^ and [Fe­(III)­Pc­(H_2_O)_2_]^+^, respectively. It is interesting to observe that, if we consider the more likely [Fe­(III)­Pc­(H_2_O)_2_]^+^ catalyst model, the formation Gibbs free energy of [Fe­(III)­Pc­(H_2_O)­(OH)]^+^ is 0.35 eV at the equilibrium potential of OER, *V* = 1.23 V, which is compatible with excellent OER activity of the iron phthalocyanine-based systems, reporting overpotentials in the range 0.3–0.4 V at the reference value of current density 10 mA/cm^2^.^110^

Further work will be focused on addressing two relevant questions: first, if other species coming from the reaction environment can effectively compete with H_2_O; second, if there is a relationship between the adsorption strength and the nature of the ligand, likely dependent on the type of interaction. In this respect, we highlight a seminal work by Schumann et al. where it was reported that adsorbate binding on single-atom alloys is strongest when the dopant and adsorbate contribute a total of ten bonding electrons, but this rule holds mainly for covalent interactions, while electrostatic cases depend more on dopant charge.^111^ Investigation of these aspects is crucial as previous studies have shown that different electrolytes solutions lead to different behavior with the redox process being sensitive to the presence of electrolyte anions, as some can poison the catalyst at modest concentrations.^71^ While a dedicated investigation is needed to fully address these points, these considerations highlight the importance of both ligand identity and the local chemical environment in governing redox and catalytic properties.

In this work, we performed quantum chemical density functional theory (DFT) calculations to investigate the nature of iron-based single-atom catalysts, using iron phthalocyanine (FePc) as a homogeneous benchmark system. Under oxidative conditions, such as during the oxygen evolution reaction (OER), the active phase is experimentally known to consist of Fe­(III), as the Fe­(II) to Fe­(III) transition occurs at relatively low oxidation potentials. However, standard quantum chemical approaches fail to reproduce this oxidation behavior, incorrectly predicting oxidation of the ligand rather than the metal center.

Our results show that explicitly including water molecules in the coordination sphere of the catalyst resolves this discrepancy, allowing the DFT calculations to correctly predict an Fe­(III) active species under oxidative conditions. Moreover, the calculated redox potential of the Fe­(II)/Fe­(III) couple aligns well with experimental values only when water molecules are coordinating the metal center. These findings underline the crucial role of water in modulating the coordination environment and, consequently, the nature and reactivity of the catalyst.

More broadly, this study provides additional evidence for the fundamental importance of coordination chemistry in single-site catalysis. The reactivity and electronic properties of such systems are strongly influenced by specific ligands present under experimental conditions, often originating from the solvent itself. This insight emphasizes the need for accurate modeling of the catalyst’s coordination environment in order to achieve reliable predictions, especially when aiming to perform high-throughput screening, identify activity descriptors, or design new catalytic systems.

Finally, our findings raise important questions regarding the nature of the active phase in iron-based heterogeneous single-atom electrocatalysts supported on carbon-based materials.

## Supplementary Material





## Data Availability

The data reported in this article can be found in the literature cited and can be provided by the authors upon reasonable request.

## Notes

Additional table, working quantities, and Cartesian coordinates of all simulated models (PDF)
Transparent Peer Review report available (PDF)
