# Supplementary material for: Role of Water in Modulating the Fe3+/Fe2+ Redox Couple in Iron-Based Complexes and Single-Atom Catalysts
Source: J Phys Chem Lett. 2025 Sep 18;16(39):10049–57. doi: 10.1021/acs.jpclett.5c02424 (PMC12498497; doi:10.1021/acs.jpclett.5c02424)
Supplement: Supplementary file 1 [file jz5c02424_si_001.pdf]

Supporting Information for

Role of Water in Modulating the Fe^{3+}/Fe^{2+} Redox Couple in Iron-Based Complexes and Single-Atom Catalysts

Alessandro Bonardi,[a/] *Shuai Xu*[b/] *Giovanni Di Liberto*[*,a] *and Gianfranco Pacchioni*[*,a]

[a]Department of Materials Science, University of Milano-Bicocca, Via Cozzi 55, 20125 Milano, Italy

[b]School of Water and Environment, Chang'an University, 710064, Xi'an, P.R. China

Corresponding Authors: Giovanni Di Liberto, giovanni.diliberto@unimib.it; Gianfranco Pacchioni, gianfranco.pacchioni@unimib.it

Table S1: Calculated properties of FePc, FePc(H_2O)$_1$ and FePc(H_2O)$_2$ complexes in oxidative conditions. Δ*E* are relative energies (in eV) with respect to the most stable state. μ_B is the magnetization of the Fe atom. Values are obtained at B3LYP level. The ground state is in bold.

		+1		+2		+3	
		**Triplet**	Quintet	**Quartet**	Sextet	**Triplet**	Quintet
	Δ*E*	**0**	1.56	**0**	1.50	**0**	0.20
FePc(H_2O)$_0$	μ_B	**2.2**	3.0	**2.0**	2.9	**2.0**	2.9
	Config.	d^6L	d^5L^{+1}	d^6L^{-1}	d^5L	d^6L^{-2}	d^5L^{-1}
		**Triplet**	Quintet	Doublet	**Quartet**	Triplet	**Quintet**
	Δ*E*	**0**	0.49	0.92	**0**	0.62	**0**
FePc(H_2O)$_1$	μ_B	**2.2**	3.9	1.0	**2.9**	2.0	**2.9**
	Config.	d^6L	d^6L	d^6L^{-1}	d^5L	d^6L^{-2}	d^5L^{-1}
		Singlet	**Triplet**	Doublet	**Quartet**	Triplet	**Quintet**
	Δ*E*	0.23	*	0.70	**0**	0.62	**0**
FePc(H_2O)$_2$	μ_B	/	*	1.0	**2.9**	1.0	**2.9**
	Config.	d^6L	*	d^6L^{-1}	d^5L	d^5L^{-1}	d^5L^{-1}

Cartesian coordinates of the optimized geometries and calculated properties of the ground state of FePc, FePc$^+$ and FePc^{2+} in vacuum, with one, two water molecules and in water. S is the vibrational entropy and ZPE is the Zero-Point Energy. Values are obtained at PBE0 level.

FePc:
ZPE = 0.534 eV
S = 1.036 * 10^{-3} eV/K
N -2.38651900 2.36091900 0.00010600
N -0.00905500 1.93074900 0.00000500
N 2.36421700 2.38326100 -0.00009400
N 1.92016100 0.00901200 -0.00000600
N 2.38651900 -2.36091900 0.00007700
N 0.00905500 -1.93074900 -0.00000500
N -2.36421700 -2.38326100 -0.00008900
N -1.92016100 -0.00901200 0.00000600
C -1.12201300 2.73561800 0.00006500
C -0.72003400 4.12452800 0.00005200
C 1.09624200 2.74611500 -0.00005100
C 0.68114600 4.13116500 -0.00003100
C -1.45007400 5.31182200 0.00009800
C -0.73477000 6.50083600 0.00005900
C 0.67303900 6.50753200 -0.00002500
C 1.39973100 5.32542700 -0.00007000
C 2.73145600 1.11929300 -0.00006100

C 4.12671500 0.71827900 -0.00005300
C 2.74192000 -1.09355300 0.00004200
C 4.13333100 -0.67937300 0.00002200
C 5.32385700 -1.39827100 0.00005500
C 5.31027700 1.44858800 -0.00009600
C 6.50954400 -0.67132300 0.00001200
C 6.50287000 0.73305200 -0.00006300
C 1.12201300 -2.73561800 0.00004300
C 0.72003400 -4.12452800 0.00002700
C -0.68114600 -4.13116500 -0.00004700
C -1.09624200 -2.74611500 -0.00005700
C -1.39973100 -5.32542700 -0.00008700
C -0.67303900 -6.50753200 -0.00005300
C 0.73477000 -6.50083600 0.00002100
C 1.45007400 -5.31182100 0.00006100
C -2.73145600 -1.11929200 -0.00004800
C -2.74192000 1.09355300 0.00006700
C -4.12671500 -0.71827900 -0.00002600
C -4.13333100 0.67937300 0.00005600
C -5.31027700 -1.44858800 -0.00006400
C -5.32385700 1.39827100 0.00010300
C -6.50287000 -0.73305200 -0.00001700
C -6.50954400 0.67132200 0.00006500
H -2.53499100 5.29230000 0.00016200
H -1.26687000 7.44767400 0.00009300
H 1.19607600 7.45940300 -0.00005400
H 2.48478600 5.31640300 -0.00013400
H 5.31438600 -2.48337000 0.00011300
H 5.29031800 2.53354500 -0.00015400
H 7.46061300 -1.19580100 0.00003700
H 7.44889600 1.26657700 -0.00009600
H -1.19607600 -7.45940300 -0.00008300
H 1.26687000 -7.44767300 0.00004700
H 2.53499100 -5.29230000 0.00011800
H -2.48478600 -5.31640300 -0.00014300
H -5.29031800 -2.53354500 -0.00012700
H -5.31438600 2.48337000 0.00016600
H -7.44889600 -1.26657800 -0.00004500
H -7.46061300 1.19580100 0.00010100
Fe 0.00000000 0.00000000 0.00000000

$FePc^{+}$:
ZPE = 0.460 eV
S = 1.186 * 10^{-3} eV/K
N -3.34713900 0.25374300 0.00005600
N -1.25264600 1.45817700 0.00000500
N 0.25374300 3.34713900 -0.00004400
N 1.45817700 1.25264600 -0.00000700
N 3.34713900 -0.25374300 0.00002600
N 1.25264600 -1.45817700 -0.00000500
N -0.25374300 -3.34713900 -0.00003800

N -1.45817700 -1.25264600 0.00000700
C -2.62192000 1.35482000 0.00003400
C -3.22262500 2.67883100 0.00002900
C -0.94409300 2.79623600 -0.00002100
C -2.16243500 3.58967400 -0.00001000
C -4.54388900 3.10043600 0.00005200
C -4.76998900 4.47805100 0.00003400
C -3.70803600 5.39044200 -0.00000600
C -2.38020700 4.95940300 -0.00002800
C 1.35482000 2.62192000 -0.00003100
C 2.67883100 3.22262500 -0.00003000
C 2.79623600 0.94409300 0.00001000
C 3.58967400 2.16243500 0.00000100
C 4.95940300 2.38020700 0.00001300
C 3.10043600 4.54388900 -0.00005000
C 5.39044200 3.70803600 -0.00000600
C 4.47805100 4.76998900 -0.00003700
C 2.62192000 -1.35482000 0.00001200
C 3.22262500 -2.67883100 0.00000300
C 2.16243500 -3.58967400 -0.00002600
C 0.94409300 -2.79623600 -0.00002700
C 2.38020700 -4.95940300 -0.00004500
C 3.70803600 -5.39044200 -0.00003300
C 4.76998900 -4.47805100 -0.00000400
C 4.54388900 -3.10043600 0.00001400
C -1.35482000 -2.62192000 -0.00001500
C -2.79623600 -0.94409300 0.00003700
C -2.67883100 -3.22262500 -0.00000200
C -3.58967400 -2.16243500 0.00003600
C -3.10043600 -4.54388900 -0.00001700
C -4.95940300 -2.38020700 0.00006000
C -4.47805200 -4.76998900 0.00000800
C -5.39044200 -3.70803600 0.00004600
H -5.35924700 2.38500600 0.00008300
H -5.78869600 4.85273100 0.00005100
H -3.92495300 6.45397400 -0.00001900
H -1.55010200 5.65767300 -0.00005900
H 5.65767300 1.55010200 0.00003700
H 2.38500600 5.35924700 -0.00007400
H 6.45397400 3.92495300 0.00000300
H 4.85273100 5.78869600 -0.00005200
H 3.92495300 -6.45397400 -0.00004800
H 5.78869600 -4.85273100 0.00000500
H 5.35924700 -2.38500600 0.00003700
H 1.55010200 -5.65767300 -0.00006800
H -2.38500600 -5.35924700 -0.00004600
H -5.65767300 -1.55010200 0.00009000
H -4.85273100 -5.78869600 -0.00000300
H -6.45397400 -3.92495300 0.00006400
Fe 0.00000000 0.00000000 0.00000000

$FePc^{2+}$:
ZPE = 0.527 eV
S = 1.049 * 10^{-3} eV/K
N -2.75119800 -1.92496100 0.00017600
N -1.89077600 0.33412200 0.00000600
N -1.92496100 2.75119800 -0.00016300
N 0.33412200 1.89077600 0.00000300
N 2.75119800 1.92496100 0.00015700
N 1.89077600 -0.33412200 -0.00000600
N 1.92496100 -2.75119800 -0.00017300
N -0.33412100 -1.89077600 -0.00000600
C -2.88700300 -0.61244100 0.00009900
C -4.19086600 0.03019400 0.00007900
C -2.50246700 1.56472900 -0.00008600
C -3.94765300 1.40762500 -0.00005500
C -5.47543200 -0.48301000 0.00015500
C -6.53050300 0.44521400 0.00009100
C -6.28826500 1.81767500 -0.00004400
C -4.97929100 2.32912000 -0.00011900
C -0.61244100 2.88700300 -0.00009400
C 0.03019400 4.19086600 -0.00008000
C 1.56472900 2.50246700 0.00008400
C 1.40762500 3.94765300 0.00005000
C 2.32911900 4.97929000 0.00010900
C -0.48301000 5.47543200 -0.00015800
C 1.81767500 6.28826400 0.00003100
C 0.44521500 6.53050300 -0.00010200
C 2.88700300 0.61244100 0.00008700
C 4.19086600 -0.03019400 0.00006100
C 3.94765300 -1.40762500 -0.00007700
C 2.50246800 -1.56472900 -0.00010100
C 4.97929100 -2.32912000 -0.00014800
C 6.28826500 -1.81767500 -0.00007700
C 6.53050300 -0.44521500 0.00006200
C 5.47543200 0.48301000 0.00013200
C 0.61244100 -2.88700200 -0.00009500
C -1.56472900 -2.50246800 0.00009800
C -0.03019400 -4.19086600 -0.00006000
C -1.40762500 -3.94765300 0.00008100
C 0.48301000 -5.47543100 -0.00012600
C -2.32912000 -4.97929100 0.00016100
C -0.44521400 -6.53050300 -0.00004700
C -1.81767500 -6.28826400 0.00009600
H -5.65778000 -1.55233200 0.00026200
H -7.55489800 0.08691300 0.00014800
H -7.12819400 2.50491400 -0.00009300
H -4.78521000 3.39635200 -0.00022600
H 3.39635200 4.78521000 0.00021300
H -1.55233200 5.65778000 -0.00026200
H 2.50491500 7.12819300 0.00007400
H 0.08691300 7.55489700 -0.00016200

H 7.12819400 -2.50491400 -0.00013200
H 7.55489800 -0.08691200 0.00011600
H 5.65778000 1.55233200 0.00024100
H 4.78521000 -3.39635200 -0.00025800
H 1.55233200 -5.65778000 -0.00023800
H -3.39635200 -4.78521000 0.00027300
H -0.08691300 -7.55489700 -0.00009700
H -2.50491500 -7.12819300 0.00015700
Fe 0.00000000 0.00000000 -0.00000100

---

1 Explicit H_2O molecule:

FePc(H_2O):
ZPE = 1.259 eV
S = 2.281 * 10^{-3} eV/K
N 3.37027700 0.00647400 -0.09714400
N 1.37071500 1.36071700 -0.06172000
N -0.00643200 3.34533900 -0.04627400
N -1.37600100 1.35550100 -0.06227300
N -3.37027800 -0.00647600 -0.09711400
N -1.37071600 -1.36072100 -0.06170600
N 0.00643200 -3.34534300 -0.04627800
N 1.37600100 -1.35550500 -0.06228300
C 2.72756300 1.15860400 -0.08940000
C 3.41659400 2.43400500 -0.10014100
C 1.15133000 2.71484900 -0.05743300
C 2.42065100 3.41779800 -0.07913000
C 4.76770000 2.76874100 -0.12700000
C 5.09233100 4.11973100 -0.13034400
C 4.09275600 5.10779000 -0.10904800
C 2.74438200 4.77146400 -0.08418300
C -1.16179600 2.71042000 -0.05771900
C -2.43378600 3.40849200 -0.07930400
C -2.73202300 1.14816100 -0.08963500
C -3.42596100 2.42088100 -0.10026200
C -4.77835200 2.75046000 -0.12692800
C -2.76269800 4.76091200 -0.08420600
C -5.10813900 4.10018900 -0.13015800
C -4.11234400 5.09207300 -0.10890800
C -2.72756400 -1.15860500 -0.08937500
C -3.41659600 -2.43400600 -0.10011300
C -2.42065400 -3.41780100 -0.07911300
C -1.15133200 -2.71485300 -0.05742400
C -2.74438600 -4.77146700 -0.08416700
C -4.09276000 -5.10779200 -0.10902200
C -5.09233400 -4.11973100 -0.13030700
C -4.76770300 -2.76874200 -0.12696200
C 1.16179700 -2.71042400 -0.05773100
C 2.73202300 -1.14816300 -0.08965800
C 2.43378700 -3.40849500 -0.07933000

C 3.42596100 -2.42088200 -0.10029500
C 2.76270000 -4.76091500 -0.08423900
C 4.77835200 -2.75046000 -0.12697500
C 4.11234600 -5.09207500 -0.10895400
C 5.10813900 -4.10018900 -0.13021100
H 5.52775800 1.99429400 -0.14564000
H 6.13528100 4.42217100 -0.15100900
H 4.38428400 6.15419600 -0.11357400
H 1.96405500 5.52547100 -0.06989700
H -5.53545000 1.97311800 -0.14550600
H -1.98526100 5.51789700 -0.06992600
H -6.15224100 4.39864000 -0.15068400
H -4.40788600 6.13735200 -0.11332900
H -4.38428900 -6.15419700 -0.11354800
H -6.13528500 -4.42217100 -0.15096400
H -5.52775900 -1.99429400 -0.14559400
H -1.96405900 -5.52547400 -0.06988900
H 1.98526300 -5.51789900 -0.06995300
H 5.53544900 -1.97311700 -0.14555800
H 4.40788800 -6.13735300 -0.11338000
H 6.15224200 -4.39863900 -0.15074700
Fe 0.00000000 -0.00000200 0.01998000
O 0.00001100 0.00003000 2.26582700
H 0.77650600 0.00751800 2.83134700
H -0.77647800 -0.00745100 2.83135600

$FePc(H_2O)^+$:
ZPE = 1.255 eV
S = 2.273 * 10^{-3} eV/K
N 0.01449000 -3.36405100 -0.08227700
N 1.35993200 -1.35347000 -0.03923100
N 3.35676000 0.01447900 -0.04068600
N 1.34820900 1.36515000 -0.04017100
N -0.01448900 3.36405200 -0.08224700
N -1.35993200 1.35347800 -0.03921500
N -3.35675800 -0.01447800 -0.04069200
N -1.34820900 -1.36515700 -0.04017900
C 1.16083400 -2.72090800 -0.07531000
C 2.43296600 -3.39806800 -0.10410700
C 2.72576000 -1.13794000 -0.04941200
C 3.41770100 -2.40175800 -0.08574800
C 2.76654600 -4.75060200 -0.14720000
C 4.11769900 -5.06728700 -0.16913100
C 5.10760900 -4.06587200 -0.15091900
C 4.77467200 -2.71914300 -0.11046100
C 2.71581200 1.16140400 -0.05003700
C 3.39686200 2.43112800 -0.08648900
C 1.13735900 2.73079400 -0.07590700
C 2.40360700 3.41892900 -0.10481100
C 2.72557400 4.77428100 -0.14781200
C 4.75104500 2.76012500 -0.11115900

C 4.07396600 5.10254600 -0.16975700
C 5.07242700 4.10966900 -0.15158800
C -1.16083300 2.72091800 -0.07528300
C -2.43296800 3.39807600 -0.10407600
C -3.41770100 2.40176300 -0.08573000
C -2.72575600 1.13794400 -0.04940400
C -4.77467200 2.71914400 -0.11044600
C -5.10761200 4.06587200 -0.15089100
C -4.11770500 5.06729100 -0.16909000
C -2.76655100 4.75060900 -0.14715800
C -2.71580800 -1.16140600 -0.05005100
C -1.13735800 -2.73080300 -0.07592800
C -3.39686200 -2.43113100 -0.08651400
C -2.40360900 -3.41893500 -0.10484100
C -4.75104400 -2.76012400 -0.11119100
C -2.72557900 -4.77428500 -0.14785400
C -5.07243000 -4.10966700 -0.15163300
C -4.07397200 -5.10254600 -0.16980700
H 1.99610800 -5.51386200 -0.16470600
H 4.42330200 -6.10837200 -0.20303700
H 6.15264500 -4.35892900 -0.17101700
H 5.52882500 -1.93968800 -0.09957900
H 1.94862200 5.53091200 -0.16524300
H 5.51185400 1.98716800 -0.10025500
H 4.37061300 6.14621900 -0.20362500
H 6.11490800 4.41168700 -0.17167300
H -6.15264900 4.35892700 -0.17099000
H -4.42331000 6.10837400 -0.20298700
H -1.99611500 5.51387200 -0.16465400
H -5.52882200 1.93968600 -0.09957400
H -5.51185100 -1.98716400 -0.10028500
H -1.94862900 -5.53091900 -0.16528800
H -6.11491200 -4.41168200 -0.17172500
H -4.37062100 -6.14621800 -0.20368300
Fe -0.00001100 -0.00000100 0.12306000
O 0.00004300 -0.00001100 2.25486100
H 0.01031700 -0.78234700 2.81560300
H -0.01023400 0.78231900 2.81561100

$FePc(H_2O)^{2+}$:
ZPE = 1.247 eV
S = 2.283 * 10^{-3} eV/K
N -0.02100200 -3.36310900 -0.07805400
N 1.34221600 -1.36421700 -0.01011600
N 3.35701700 -0.02095000 -0.01821000
N 1.35915400 1.34732600 -0.01112200
N 0.02099800 3.36311000 -0.07802400
N -1.34222100 1.36421100 -0.01010200
N -3.35702400 0.02095000 -0.01820800
N -1.35915900 -1.34731900 -0.01113200
C 1.13182700 -2.73081000 -0.06529500

C 2.39824200 -3.42382500 -0.11043400
C 2.71238500 -1.16630600 -0.02921000
C 3.39338600 -2.43859700 -0.08432800
C 2.71136700 -4.77525500 -0.17663400
C 4.06726700 -5.10765500 -0.21286800
C 5.06311000 -4.12186500 -0.18680300
C 4.74304600 -2.76407700 -0.12388200
C 2.72672000 1.13237700 -0.02942800
C 3.42359400 2.39608500 -0.08387800
C 1.16584800 2.71642900 -0.06550700
C 2.44081300 3.39363500 -0.10999500
C 2.77073700 4.74107700 -0.17555100
C 4.77721600 2.70477000 -0.12276600
C 4.13069000 5.05660800 -0.21115600
C 5.11417100 4.05850100 -0.18507600
C -1.13183000 2.73080400 -0.06527000
C -2.39824300 3.42382200 -0.11040400
C -3.39339000 2.43859700 -0.08430600
C -2.71239300 1.16630500 -0.02919800
C -4.74305000 2.76408100 -0.12385700
C -5.06310800 4.12187100 -0.18676800
C -4.06726300 5.10765800 -0.21282400
C -2.71136400 4.77525400 -0.17659400
C -2.72672800 -1.13237500 -0.02943700
C -1.16585200 -2.71642200 -0.06553100
C -3.42359800 -2.39608400 -0.08389900
C -2.44081300 -3.39363000 -0.11002600
C -4.77722000 -2.70477200 -0.12279000
C -2.77073400 -4.74107300 -0.17559500
C -5.11417000 -4.05850300 -0.18511300
C -4.13068500 -5.05660700 -0.21120300
H 1.93588600 -5.53314500 -0.20105600
H 4.35930200 -6.15166200 -0.26449700
H 6.10520100 -4.42340700 -0.21849400
H 5.50865400 -1.99602300 -0.10751100
H 2.00475500 5.50856500 -0.19996600
H 5.53321000 1.92725000 -0.10636900
H 4.43571300 6.09691800 -0.26228600
H 6.15994900 4.34705400 -0.21625900
H -6.10519900 4.42341600 -0.21845600
H -4.35929500 6.15166700 -0.26444500
H -1.93588000 5.53314100 -0.20101000
H -5.50866000 1.99603000 -0.10749300
H -5.53321600 -1.92725500 -0.10638500
H -2.00474900 -5.50855700 -0.20001800
H -6.15994700 -4.34705900 -0.21629900
H -4.43570600 -6.09691800 -0.26234400
Fe 0.00000600 -0.00000100 0.16913400
O 0.00001600 -0.00001000 2.29405700
H 0.00248000 -0.77985100 2.86074500
H -0.00250500 0.77982600 2.86075200

----

2 Explicit H_2O molecules:

$FePc(H_2O)_2$:
ZPE = 1.950 eV
S = 3.907 * 10^{-3} eV/K
N 2.39534300 2.35632400 0.00011600
N 0.01568400 1.91869500 -0.00006000
N -2.35651500 2.39538000 -0.00017000
N -1.95431700 0.01614600 -0.00013400
N -2.39540100 -2.35631100 -0.00001600
N -0.01575100 -1.91869300 -0.00005200
N 2.35645700 -2.39538900 -0.00001900
N 1.95427400 -0.01614400 -0.00001400
C 1.13277500 2.72422500 0.00004900
C 0.73304600 4.12278800 0.00005400
C -1.08801900 2.74241000 -0.00008700
C -0.66533300 4.13420300 -0.00000100
C 1.46575200 5.30464400 0.00011900
C 0.75505800 6.50104700 0.00014600
C -0.64858700 6.51247500 0.00010100
C -1.37868700 5.32779800 0.00003000
C -2.74527900 1.12924400 -0.00016200
C -4.13795800 0.73556100 -0.00010300
C -2.76325000 -1.08402600 -0.00005400
C -4.14934100 -0.66793800 -0.00013500
C -5.34735700 -1.38047700 -0.00018000
C -5.32434500 1.46725700 -0.00007800
C -6.52806100 -0.65083900 -0.00016200
C -6.51668000 0.75670800 -0.00011700
C -1.13283200 -2.72421400 -0.00000100
C -0.73311000 -4.12277900 0.00003300
C 0.66526900 -4.13420800 0.00006000
C 1.08796200 -2.74241800 -0.00001600
C 1.37861200 -5.32781100 0.00013900
C 0.64850000 -6.51248100 0.00017800
C -0.75514600 -6.50103700 0.00014500
C -1.46582700 -5.30462800 0.00006800
C 2.74524100 -1.12924800 -0.00003300
C 2.76321200 1.08403300 0.00006700
C 4.13790900 -0.73556400 0.00006700
C 4.14929200 0.66794400 0.00002900
C 5.32430700 -1.46725700 0.00014000
C 5.34731900 1.38047900 0.00002300
C 6.51663700 -0.75670900 0.00014500
C 6.52801800 0.65084300 0.00009200
H 2.55082000 5.28060000 0.00016200
H 1.29270500 7.44491500 0.00020500
H -1.17078200 7.46497300 0.00011900

H -2.46400400 5.32131100 -0.00001200
H -5.34263000 -2.46582100 -0.00017500
H -5.30214100 2.55237600 -0.00006800
H -7.48152400 -1.17137200 -0.00016900
H -7.46164000 1.29250700 -0.00012000
H 1.17068500 -7.46498400 0.00023100
H -1.29280200 -7.44490000 0.00018600
H -2.55089500 -5.28056900 0.00005700
H 2.46392900 -5.32133900 0.00015100
H 5.30210600 -2.55237700 0.00014300
H 5.34259500 2.46582300 0.00001000
H 7.46159800 -1.29250700 0.00017500
H 7.48148200 1.17137500 0.00011400
Fe -0.00005000 0.00000100 -0.00003000
O -0.00011600 -0.00000600 2.27468400
H 0.00787400 0.77701900 2.83914300
H -0.00014100 -0.77708100 2.83912900
O 0.00015200 -0.00001000 -2.27479000
H 0.00715400 0.77701500 -2.83926200
H -0.00638700 -0.77704500 -2.83925400

$FePc(H_2O)_2^+$:
ZPE = 1.923 eV
S = 4.209 * 10^{-3} eV/K
N 2.37449100 2.38002300 -0.04613500
N -0.00224400 1.93257500 0.00035000
N -2.38005200 2.37446300 0.04612000
N -1.93264800 -0.00224300 -0.00035000
N -2.37449200 -2.38002200 -0.04610500
N 0.00224400 -1.93257500 0.00036900
N 2.38005200 -2.37446300 0.04612700
N 1.93264800 0.00224300 -0.00035500
C 1.10982700 2.74552700 -0.02849200
C 0.69579500 4.12920100 -0.02031300
C -1.11623900 2.74290300 0.02871200
C -0.70547800 4.12754700 0.02016700
C 1.42066200 5.31884200 -0.04063700
C 0.69572900 6.50293600 -0.02044200
C -0.71106100 6.50127100 0.01952800
C -1.43317700 5.31546400 0.04008700
C -2.74556700 1.10979000 0.02848500
C -4.12926900 0.69579000 0.02030900
C -2.74294300 -1.11620100 -0.02870500
C -4.12761400 -0.70547300 -0.02016600
C -5.31551800 -1.43317200 -0.04008500
C -5.31889700 1.42065800 0.04062700
C -6.50133600 -0.71105600 -0.01953200
C -6.50300100 0.69572300 0.02043300
C -1.10982700 -2.74552700 -0.02846500
C -0.69579500 -4.12920100 -0.02028200
C 0.70547800 -4.12754700 0.02019200

C 1.11624000 -2.74290300 0.02872700
C 1.43317600 -5.31546500 0.04011300
C 0.71106000 -6.50127100 0.01956300
C -0.69573000 -6.50293500 -0.02040000
C -1.42066300 -5.31884200 -0.04059600
C 2.74556800 -1.10979000 0.02848100
C 2.74294300 1.11620100 -0.02872600
C 4.12926900 -0.69579000 0.02029000
C 4.12761400 0.70547400 -0.02019500
C 5.31889800 -1.42065700 0.04060400
C 5.31551700 1.43317300 -0.04012800
C 6.50300100 -0.69572200 0.02039500
C 6.50133500 0.71105700 -0.01958000
H 2.50492000 5.30885300 -0.07177200
H 1.22088600 7.45292500 -0.03572900
H -1.23848100 7.45001200 0.03450900
H -2.51740900 5.30289700 0.07119500
H -5.30295500 -2.51740500 -0.07118800
H -5.30891100 2.50491700 0.07175800
H -7.45007500 -1.23847900 -0.03451200
H -7.45298800 1.22088500 0.03571600
H 1.23847900 -7.45001200 0.03454500
H -1.22088800 -7.45292500 -0.03568000
H -2.50492100 -5.30885200 -0.07172700
H 2.51740800 -5.30289800 0.07121500
H 5.30891300 -2.50491500 0.07174300
H 5.30295300 2.51740500 -0.07124100
H 7.45298800 -1.22088300 0.03567400
H 7.45007400 1.23848000 -0.03457200
Fe -0.00000100 0.00000100 0.00000300
O -0.00001400 0.00002700 2.21919000
H 0.54558700 0.55732500 2.78187900
H -0.54540800 -0.55746600 2.78188600
O -0.00000100 -0.00001200 -2.21918700
H -0.55193200 0.55101000 -2.78188300
H 0.55192800 -0.55104200 -2.78187700

$FePc(H_2O)_2^{2+}$:
ZPE = 1.917 eV
S = 5.233 * 10^{-3} eV/K
N 2.37779600 2.37580700 -0.06002900
N 0.00080900 1.92768900 0.00039500
N -2.37582900 2.37777700 0.06001300
N -1.92775800 0.00081000 -0.00040100
N -2.37779600 -2.37580800 -0.06000000
N -0.00080900 -1.92769000 0.00041500
N 2.37583000 -2.37777700 0.06001800
N 1.92775800 -0.00081100 -0.00040500
C 1.11307900 2.74246500 -0.03635100
C 0.70192000 4.13014900 -0.02680000
C -1.11081300 2.74338900 0.03615600

C -0.69854400 4.13073300 0.02536400
C 1.43000900 5.31188300 -0.05376000
C 0.70250500 6.50413100 -0.02773000
C -0.69723700 6.50471400 0.02409900
C -1.42569100 5.31307200 0.05122800
C -2.74250200 1.11304100 0.03634100
C -4.13020500 0.70191700 0.02679600
C -2.74342500 -1.11077400 -0.03615100
C -4.13078800 -0.69854200 -0.02536000
C -5.31311700 -1.42568900 -0.05121800
C -5.31192900 1.43000400 0.05375500
C -6.50477000 -0.69723400 -0.02409000
C -6.50418800 0.70249800 0.02773100
C -1.11307900 -2.74246500 -0.03632500
C -0.70192000 -4.13015000 -0.02677100
C 0.69854500 -4.13073400 0.02538600
C 1.11081400 -2.74338900 0.03617100
C 1.42569200 -5.31307200 0.05125100
C 0.69723800 -6.50471400 0.02412900
C -0.70250500 -6.50413100 -0.02769300
C -1.43000800 -5.31188300 -0.05372300
C 2.74250300 -1.11304100 0.03633600
C 2.74342500 1.11077300 -0.03617300
C 4.13020600 -0.70191700 0.02677600
C 4.13078800 0.69854100 -0.02539000
C 5.31193000 -1.43000400 0.05372900
C 5.31311700 1.42568800 -0.05126500
C 6.50418800 -0.70249800 0.02768800
C 6.50477000 0.69723300 -0.02414300
H 2.51389900 5.30458600 -0.09433400
H 1.23076600 7.45198600 -0.04797800
H -1.22474300 7.45300800 0.04347500
H -2.50958800 5.30667700 0.09181100
H -5.30672600 -2.50958600 -0.09179500
H -5.30463800 2.51389500 0.09432400
H -7.45306100 -1.22474500 -0.04346200
H -7.45204100 1.23076300 0.04798000
H 1.22474300 -7.45300800 0.04350500
H -1.23076600 -7.45198600 -0.04793600
H -2.51389900 -5.30458700 -0.09429200
H 2.50958900 -5.30667700 0.09182700
H 5.30464000 -2.51389500 0.09430500
H 5.30672500 2.50958400 -0.09185000
H 7.45204200 -1.23076300 0.04793100
H 7.45306100 1.22474400 -0.04352800
Fe 0.00000000 0.00000000 0.00000100
O -0.00001900 0.00003200 2.22008500
H 0.54423500 0.55430200 2.78914900
H -0.54415100 -0.55435100 2.78915600
O -0.00000100 -0.00001200 -2.22008600
H -0.54905000 0.54950200 -2.78915600

H 0.54904600 -0.54953300 -2.78915200

---

4 Explicit H_2O molecules:

$FePc(H_2O)_4$:
ZPE = 3.356 eV
S = 6.520 * 10^{-3} eV/K
N 0.19599900 -3.34231400 -0.00014100
N 1.32013300 -1.19935900 0.00032500
N 3.16307900 0.37486600 0.00034400
N 1.04456600 1.53884900 -0.00015200
N -0.55236300 3.34966400 -0.00015500
N -1.66795800 1.20109500 -0.00007300
N -3.50951200 -0.36687500 -0.00007300
N -1.39138100 -1.52509600 -0.00000900
C 1.26203400 -2.57576000 0.00011300
C 2.60931400 -3.12340500 0.00027400
C 2.64899600 -0.84021000 0.00055600
C 3.48412000 -2.02962900 0.00062100
C 3.08728300 -4.42967700 0.00005600
C 4.46688800 -4.61084500 0.00023500
C 5.34443300 -3.51468100 0.00059700
C 4.86526900 -2.20842600 0.00076900
C 2.41033600 1.47453000 -0.00004200
C 2.96135700 2.80777100 -0.00005900
C 0.68133400 2.85359400 -0.00011000
C 1.86152100 3.68406000 -0.00010200
C 2.04005700 5.06788700 -0.00008900
C 4.26978000 3.29489000 -0.00001200
C 3.34069700 5.54815000 -0.00004900
C 4.44307600 4.67057200 -0.00001300
C -1.61405100 2.58074200 -0.00021100
C -2.96415200 3.12735500 -0.00042300
C -3.83341100 2.03409600 -0.00042600
C -2.99892300 0.84059500 -0.00020700
C -5.21205000 2.20721500 -0.00063000
C -5.69430100 3.51348800 -0.00082600
C -4.82111100 4.61127500 -0.00081700
C -3.44023300 4.43270500 -0.00061800
C -2.75369100 -1.45696500 0.00004100
C -1.03013500 -2.84242600 -0.00011600
C -3.30824900 -2.79251600 0.00000800
C -2.21468200 -3.67176000 -0.00012100
C -4.61796000 -3.26884800 0.00006000
C -2.39762500 -5.05326300 -0.00019600
C -4.79926900 -4.64495700 -0.00001100
C -3.70230600 -5.52687300 -0.00014100
H 2.39705500 -5.26713900 -0.00023000
H 4.87618700 -5.61684700 0.00008800

H 6.41581500 -3.69307200 0.00073600
H 5.53395500 -1.35304200 0.00104600
H 1.18210000 5.73254900 -0.00011000
H 5.11409600 2.61250500 0.00002900
H 3.51878800 6.61954000 -0.00004200
H 5.44721400 5.08484300 0.00001600
H -6.76618300 3.68925800 -0.00099200
H -5.23261500 5.61642700 -0.00097200
H -2.75177700 5.27166700 -0.00061000
H -5.87588100 1.34878000 -0.00063800
H -5.45549100 -2.57874100 0.00015800
H -1.54346300 -5.72274400 -0.00029900
H -5.80553800 -5.05405500 0.00003200
H -3.88540600 -6.59755200 -0.00019800
Fe -0.17464100 -0.00117400 0.00006900
O -0.10473100 0.04415500 2.25071400
H 0.81784300 0.00812400 2.58826100
H -0.49852400 0.83705800 2.62573000
O -0.10301300 0.04245500 -2.25054700
H 0.82025800 0.00520500 -2.58609900
H -0.49506600 0.83560800 -2.62684000
O 2.52253600 -0.12704300 2.99779500
H 3.02634000 0.28746000 2.28426800
H 2.74706900 -1.06095400 2.92432100
O 2.52427000 -0.12994000 -2.99688200
H 3.02824800 0.28452800 -2.28344600
H 2.74912300 -1.06380800 -2.92376000

$FePc(H_2O)_4^{+}$:
ZPE = 3.365 eV
S = 6.761 * 10^{-3} eV/K
N 2.52219400 -2.37528700 -0.07358400
N 0.14444700 -1.93535600 -0.11060800
N -2.23252400 -2.37701800 -0.11940400
N -1.79781700 0.00115500 0.00042100
N -2.22982700 2.37982100 0.11995600
N 0.14664700 1.93546500 0.11118600
N 2.52486500 2.37276100 0.07314500
N 2.08081200 -0.00100500 -0.00045900
C 1.25562300 -2.74220300 -0.10207300
C 0.84689100 -4.12854600 -0.13281600
C -0.96932800 -2.74635600 -0.13218500
C -0.55481000 -4.13241700 -0.15361400
C 1.57809500 -5.31369300 -0.14257100
C 0.85962500 -6.50201700 -0.17410800
C -0.54667400 -6.50643500 -0.19642000
C -1.27552500 -5.32355100 -0.18657900
C -2.60282500 -1.10907600 -0.06804600
C -3.98729300 -0.69914500 -0.04309300
C -2.60156100 1.11229900 0.06855000
C -3.98649700 0.70397600 0.04305900

C -5.17901400 1.42790800 0.08643800
C -5.18063100 -1.42168800 -0.08696800
C -6.36368000 0.70684200 0.04211500
C -6.36447800 -0.69924600 -0.04314200
C -0.96620900 2.74771800 0.13284900
C -0.55013600 4.13331700 0.15425300
C 0.85155100 4.12787200 0.13318700
C 1.25872200 2.74107600 0.10224900
C 1.58409000 5.31219200 0.14280800
C 0.86695900 6.50132200 0.17452000
C -0.53932800 6.50731800 0.19709300
C -1.26951000 5.32524900 0.18734800
C 2.88963400 1.10987300 0.03097000
C 2.88838200 -1.11279600 -0.03183600
C 4.27810200 0.69764500 0.01783500
C 4.27731400 -0.70212500 -0.01900500
C 5.46426400 1.42366900 0.03700600
C 5.46266200 -1.42947600 -0.03834900
C 6.65091700 0.69870500 0.01757000
C 6.65012700 -0.70583900 -0.01912400
H 2.66254900 -5.29731100 -0.12515900
H 1.38989200 -7.44914900 -0.18248100
H -1.06997800 -7.45723500 -0.22291400
H -2.36024500 -5.31893600 -0.20735500
H -5.16889000 2.51061100 0.15747300
H -5.17173500 -2.50440100 -0.15800300
H -7.31276700 1.23281500 0.07490600
H -7.31416100 -1.22411400 -0.07633100
H 1.39828800 7.44786000 0.18281200
H -1.06155900 7.45870300 0.22370900
H -2.35423000 5.32184500 0.20829200
H 2.66852100 5.29459200 0.12515900
H 5.45346700 2.50795300 0.06501200
H 5.45064700 -2.51374800 -0.06631900
H 7.60014000 1.22521300 0.03101200
H 7.59875900 -1.23341000 -0.03271600
Fe 0.14573500 0.00007300 0.00017700
O 0.11625400 0.21731900 -2.19280700
H 0.31160900 -0.53468500 -2.75970300
H -0.69381100 0.66126900 -2.54693900
O 0.11786000 -0.21756800 2.19313400
H -0.68985200 -0.66559300 2.54753700
H 0.30951500 0.53534100 2.76009200
O -2.09835600 1.49850200 -2.96694600
H -2.93376500 1.04902900 -2.79996500
H -2.17665700 2.34149400 -2.50571900
O -2.09596500 -1.50043600 2.96717800
H -2.17747900 -2.34251900 2.50484900
H -2.93005700 -1.04801600 2.80162600

----

6 Explicit H_2O molecules:

$FePc(H_2O)_6$:
ZPE = 4.747 eV
S = 9.810 * 10^{-3} eV/K
N -2.37007400 2.56137000 -0.00070400
N -1.95241100 0.17538700 -0.00003500
N -2.37686200 -2.21417000 0.00038200
N -0.00084500 -1.75744600 0.00024700
N 2.37478600 -2.21618900 -0.00004900
N 1.95235700 0.17373000 0.00004200
N 2.37201300 2.55937300 0.00014100
N 0.00076600 2.09016000 -0.00033700
C -2.75037600 1.28467500 -0.00024000
C -4.13045500 0.88175100 0.00016400
C -2.75644100 -0.93321700 0.00041600
C -4.13449500 -0.52852900 0.00059800
C -5.32600600 1.60646300 0.00022200
C -6.51158800 0.89145800 0.00072900
C -6.51651000 -0.52075200 0.00117500
C -5.33708300 -1.24556300 0.00112100
C -1.11238500 -2.57199700 0.00018700
C -0.70087600 -3.96683900 -0.00016200
C 1.11005100 -2.57296600 -0.00003000
C 0.69732200 -3.96744400 -0.00031900
C 1.41961300 -5.15582000 -0.00070100
C -1.42420000 -5.15458800 -0.00039900
C 0.69890500 -6.34600700 -0.00093700
C -0.70452400 -6.34539800 -0.00078900
C 2.75544700 -0.93555800 0.00012500
C 4.13382600 -0.53203900 0.00042100
C 4.13098200 0.87825800 0.00056500
C 2.75125700 1.28232800 0.00026600
C 5.32715500 1.60196000 0.00088800
C 6.51212500 0.88595000 0.00107600
C 6.51585300 -0.52627000 0.00094300
C 5.33582100 -1.25008600 0.00061600
C 1.11307400 2.90490400 -0.00020900
C -1.11079400 2.90581700 -0.00072900
C 0.69880900 4.30356300 -0.00057600
C -0.69538700 4.30413800 -0.00096600
C 1.42426700 5.48703400 -0.00059300
C -1.41987200 5.48820200 -0.00138400
C 0.70353600 6.67937200 -0.00102600
C -0.69816400 6.67995100 -0.00141900
H -5.30835200 2.69156900 -0.00011000
H -7.45952700 1.42147900 0.00079700
H -7.46818600 -1.04430600 0.00157600
H -5.33193100 -2.33122600 0.00145400
H 2.50495400 -5.14327900 -0.00082200

H -2.50953000 -5.14109800 -0.00028900
H 1.22855400 -7.29412500 -0.00125400
H -1.23499500 -7.29305700 -0.00099600
H 7.46051600 1.41516300 0.00132800
H 7.46708600 -1.05062800 0.00110300
H 5.32976600 -2.33574400 0.00050800
H 5.31041500 2.68707900 0.00097900
H 2.50926800 5.47041200 -0.00027900
H -2.50488600 5.47247600 -0.00167300
H 1.23419700 7.62704700 -0.00105900
H -1.22804600 7.62806000 -0.00175100
Fe -0.00001800 0.17256500 -0.00003300
O -0.00007600 0.11460200 2.21394200
H -0.78234700 -0.35788900 2.56693800
H 0.78214600 -0.35775300 2.56710500
O -0.00008200 0.11331700 -2.21416500
H -0.78209600 -0.35999100 -2.56661700
H 0.78246500 -0.35890200 -2.56683500
O -2.28805800 -1.24826300 2.99930000
H -2.41075900 -1.94468800 2.34166200
H -3.01347500 -0.63944100 2.82220900
O -2.28717000 -1.25164900 -2.99837800
H -2.41016000 -1.94650000 -2.33909900
H -3.01330300 -0.64296600 -2.82382400
O 2.28826500 -1.25001500 -2.99913000
H 3.01454500 -0.64225500 -2.82197300
H 2.40958000 -1.94635000 -2.34112100
O 2.28823600 -1.24858200 2.99932300
H 3.01452400 -0.64108800 2.82128000
H 2.40967300 -1.94596100 2.34247700

$FePc(H_2O)_6^+$:
ZPE = 4.747 eV
S = 1.033 * 10^{-2} eV/K
N 2.37682100 -2.37093000 -0.18477400
N 0.00066400 -1.93909700 -0.18677700
N -2.37528200 -2.37222800 -0.18451800
N -1.94062100 -0.00019200 0.00004900
N -2.37662000 2.37161600 0.18440900
N -0.00044300 1.93979300 0.18678400
N 2.37548000 2.37286200 0.18520400
N 1.94075100 0.00083800 0.00006200
C 1.11027900 -2.74215800 -0.22903200
C 0.70215200 -4.12901300 -0.30989500
C -1.10858700 -2.74278400 -0.22894100
C -0.69967300 -4.12939900 -0.30984800
C 1.42783600 -5.31438000 -0.38025100
C 0.70461100 -6.49945800 -0.44808000
C -0.70079700 -6.49985100 -0.44804100
C -1.42468800 -5.31518300 -0.38016700
C -2.74381900 -1.10789000 -0.09722500

C -4.13187200 -0.69965400 -0.06083000
C -2.74445900 1.10708400 0.09727700
C -4.13227600 0.69806600 0.06068100
C -5.32205700 1.42006700 0.12507000
C -5.32122800 -1.42231800 -0.12556700
C -6.50808000 0.69912900 0.06159400
C -6.50767100 -0.70204500 -0.06243600
C -1.11013200 2.74287600 0.22881200
C -0.70196700 4.12969800 0.30991400
C 0.69986400 4.13004900 0.31031900
C 1.10874300 2.74343100 0.22935300
C 1.42489200 5.31580100 0.38098700
C 0.70101600 6.50048300 0.44876800
C -0.70439400 6.50013100 0.44836200
C -1.42763400 5.31508600 0.38016900
C 2.74396200 1.10857800 0.09764200
C 2.74461100 -1.10637900 -0.09745600
C 4.13202300 0.70034200 0.06093500
C 4.13243100 -0.69733400 -0.06100800
C 5.32138300 1.42301700 0.12554200
C 5.32220100 -1.41929400 -0.12598200
C 6.50782300 0.70278500 0.06182500
C 6.50822800 -0.69834700 -0.06264700
H 2.51265400 -5.30435600 -0.38505100
H 1.23219200 -7.44648000 -0.50483600
H -1.22785000 -7.44716800 -0.50477300
H -2.50951200 -5.30578500 -0.38491100
H -5.31239100 2.50041000 0.22586300
H -5.31091400 -2.50265500 -0.22636200
H -7.45726700 1.22379800 0.10936800
H -7.45655500 -1.22723800 -0.11050200
H 1.22807900 7.44777900 0.50576700
H -1.23196200 7.44716500 0.50505100
H -2.51245400 5.30509500 0.38460900
H 2.50971300 5.30636000 0.38605800
H 5.31107900 2.50332300 0.22665900
H 5.31251600 -2.49960600 -0.22710600
H 7.45670500 1.22799400 0.10975200
H 7.45741300 -1.22297700 -0.11089200
Fe 0.00010300 0.00035900 0.00006200
O -0.00069400 0.27250400 -2.18268500
H 0.78482200 0.76818500 -2.50255600
H -0.78661000 0.76798700 -2.50210600
O 0.00048700 -0.27145100 2.18244400
H -0.78638500 -0.76536700 2.50165800
H 0.78494000 -0.76985500 2.50092000
O -2.18460500 1.73229200 -2.91548100
H -2.96144800 1.26643100 -3.23901800
H -2.50931600 2.33809200 -2.23979300
O -2.18004300 -1.73772900 2.91546600
H -2.50479700 -2.34203000 2.23845400

H -2.95781000 -1.27786300 3.24528100
O 2.17942700 -1.73930000 2.91444200
H 2.50724800 -2.34177000 2.23727300
H 2.95474500 -1.27532200 3.24426800
O 2.18306500 1.73414400 -2.91524200
H 2.50828700 2.33977900 -2.23966400
H 2.95953600 1.26758700 -3.23868500

---

8 Explicit H_2O molecules:

$FePc(H_2O)_8$:
ZPE = 6.155 eV
S = 1.351 * 10^{-2} eV/K
N 3.13087600 -1.79934500 -0.14511400
N 2.24130100 0.45020700 -0.15316800
N 2.17814200 2.86915400 -0.13293600
N -0.05693000 1.95109100 0.01000500
N -2.47052000 1.93481800 0.16976300
N -1.58977200 -0.32126500 0.10117500
N -1.51792400 -2.74372200 0.04106900
N 0.71452800 -1.81546000 -0.06905600
C 3.24432100 -0.47075900 -0.16684300
C 4.52077300 0.19979400 -0.18081100
C 2.80424100 1.69898000 -0.16196500
C 4.24003300 1.57767900 -0.18301800
C 5.83426800 -0.27240900 -0.18782000
C 6.85340400 0.66769400 -0.19478600
C 6.57258000 2.04885700 -0.19658700
C 5.26874700 2.52079500 -0.19114400
C 0.87711800 2.96869700 -0.05597800
C 0.19606500 4.25385800 -0.01688800
C -1.29694200 2.52973600 0.10912500
C -1.17074700 3.98019500 0.08880400
C -2.11352800 5.00051900 0.15028400
C 0.66639800 5.56114000 -0.06520600
C -1.64588500 6.31061400 0.09938100
C -0.27393400 6.58578000 -0.00689200
C -2.59108800 0.59959600 0.16641200
C -3.86072100 -0.06894400 0.21085400
C -3.57906600 -1.45098200 0.15443600
C -2.14999400 -1.56844800 0.09153300
C -4.60781200 -2.39754200 0.21484600
C -5.90576200 -1.93308400 0.34850500
C -6.18694800 -0.55042800 0.41700200
C -5.17415600 0.39361000 0.34310800
C -0.21578200 -2.83900100 -0.02082600
C 1.96070900 -2.39309900 -0.12161600
C 0.46797100 -4.12419800 -0.05764700
C 1.83467700 -3.84459300 -0.11593700

C -0.00120600 -5.43187000 -0.03848700
C 2.78206900 -4.85983200 -0.15864600
C 0.94407000 -6.45335600 -0.08060900
C 2.31675800 -6.17190800 -0.13962700
H 6.03656200 -1.33884600 -0.19041800
H 7.88859300 0.33900600 -0.20052800
H 7.39853400 2.75437200 -0.20269400
H 5.04217900 3.58209400 -0.19178700
H -3.17130100 4.77279100 0.24040800
H 1.73004600 5.76065600 -0.14502600
H -2.35089200 7.13543000 0.14666300
H 0.05721600 7.61967300 -0.04158800
H -6.72706900 -2.64119800 0.40659100
H -7.21765500 -0.22620500 0.52660500
H -5.38037200 1.45826200 0.39608900
H -4.38144200 -3.45822000 0.16806000
H -1.06586200 -5.63709700 0.00852500
H 3.84066000 -4.62581400 -0.20771400
H 0.61553200 -7.48853000 -0.06778100
H 3.02594100 -6.99377300 -0.17206600
Fe 0.32947000 0.06693700 -0.01877300
O 0.51905200 0.13251300 2.14302500
H 1.13713500 -0.46940300 2.59541600
H -0.27048000 0.34462500 2.69500200
O 0.05623200 0.08527200 -2.24185200
H -0.60154100 0.75821700 -2.47820400
H -0.34297600 -0.76703100 -2.55127900
O -1.74507800 0.90947100 3.32245300
H -2.07052500 1.58260700 2.71589700
H -2.45133100 0.23022900 3.34789000
O -2.26479000 1.80770600 -2.74586400
H -2.13948900 2.64021600 -3.21052300
H -2.56380900 2.04557100 -1.85072500
O -1.34586100 -2.07305500 -3.01888300
H -1.48124400 -2.67470600 -2.27825400
H -2.21124100 -1.62335400 -3.13881700
O 2.76982600 -1.26250600 2.92915400
H 3.01833700 -1.81637300 2.17785700
H 3.34562400 -0.49514400 2.84307100
O -3.71789300 -1.02254400 3.25604800
H -3.34750100 -1.74634700 2.73857700
H -4.43957100 -0.70508800 2.69933600
O -3.58432900 -0.56172100 -3.37266400
H -3.21551400 0.32960900 -3.23348500
H -3.84172200 -0.58858600 -4.29824600

$FePc(H_2O)_8^+$:
ZPE = 6.151 eV
S = 1.391 * 10^{-2} eV/K
N 3.04417700 -1.88237200 -0.07539200
N 2.24522600 0.39579500 -0.04004100

N 2.30082900 2.81055200 -0.00528200
N 0.01806500 2.01560100 0.00053900
N -2.39733400 2.07392700 -0.00466700
N -1.59972600 -0.20727200 0.02209700
N -1.65297200 -2.62445300 0.00779000
N 0.62762000 -1.82346900 -0.07198700
C 3.21175700 -0.57382200 -0.04645100
C 4.52082600 0.04815500 -0.00884000
C 2.86311100 1.61972300 -0.00954300
C 4.30039200 1.43168700 0.01292700
C 5.80684400 -0.48212100 -0.00096500
C 6.86549800 0.41914400 0.03456100
C 6.64432600 1.80628000 0.05818800
C 5.35790200 2.33444900 0.04600200
C 0.99368300 2.97945500 -0.01230600
C 0.37350900 4.28812100 -0.03878700
C -1.20276500 2.63759600 -0.00699400
C -1.01196500 4.07351800 -0.04027000
C -1.90958100 5.13759000 -0.05952900
C 0.90916100 5.57243200 -0.06125600
C -1.37542200 6.42068100 -0.08464300
C 0.01380700 6.63511000 -0.08611900
C -2.56346000 0.76167900 0.05135000
C -3.86664900 0.13986400 0.13098600
C -3.64615300 -1.24497900 0.13056200
C -2.21502100 -1.43273300 0.04962400
C -4.70303400 -2.14843500 0.21718300
C -5.98632300 -1.62389700 0.32223900
C -6.20833400 -0.23283500 0.33577300
C -5.15231900 0.66775300 0.23584600
C -0.34812400 -2.79266900 -0.05999500
C 1.84710500 -2.44442000 -0.11274600
C 0.27360000 -4.09802100 -0.12645500
C 1.65874700 -3.87834700 -0.15869800
C -0.25606200 -5.38606600 -0.15659100
C 2.56054000 -4.93746900 -0.22340100
C 0.64349000 -6.44269000 -0.22190200
C 2.03194100 -6.22171700 -0.25516700
H 5.96785200 -1.55476300 -0.02750100
H 7.88542300 0.04726400 0.04073300
H 7.49819300 2.47618800 0.08352400
H 5.17563700 3.40369700 0.06023400
H -2.98076600 4.96406900 -0.04797800
H 1.98308800 5.72550600 -0.05843700
H -2.04136200 7.27802300 -0.09940400
H 0.39034600 7.65313100 -0.10399100
H -6.83720300 -2.29419500 0.39675500
H -7.22479000 0.13880400 0.42268900
H -5.31332100 1.74094700 0.24689700
H -4.51998000 -3.21820300 0.20387700
H -1.32861200 -5.54710300 -0.12749000

H 3.62981200 -4.75636100 -0.25276500
H 0.27142500 -7.46222200 -0.24786000
H 2.70053000 -7.07530400 -0.30807800
Fe 0.32544200 0.09682300 -0.00369000
O 0.41900800 0.08701200 2.15718400
H 0.99703800 -0.59299700 2.56100800
H -0.45034400 0.15856100 2.62713700
O 0.26699100 0.16122200 -2.23594400
H -0.40255600 0.79141400 -2.55123400
H -0.00053500 -0.71720000 -2.61972700
O -1.94806500 0.59712600 3.13319500
H -1.99021800 1.21913000 3.86357500
H -2.74088000 0.02372200 3.21817400
O -2.03390900 1.74327800 -2.96195700
H -1.97367600 2.42549600 -3.63814400
H -2.39644700 2.16900900 -2.17057800
O -0.76951300 -2.06186900 -3.21440000
H -0.72085600 -2.87822400 -2.70907400
H -1.70716000 -1.77095600 -3.17443100
O 2.32908500 -1.65883500 3.02319300
H 2.81406400 -2.05561200 2.28994800
H 2.99321100 -1.19385300 3.54127000
O -4.24967500 -0.82059900 3.28799900
H -4.19329500 -1.74645000 3.03190400
H -4.85586200 -0.43499800 2.64366500
O -3.13818500 -0.80761200 -2.97606800
H -2.87652600 0.11620600 -3.13586800
H -3.90699400 -0.97416600 -3.52739100

---

10 Explicit H_2O molecules:

$FePc(H_2O)_{10}$:
ZPE = 7.575 eV
S = 1.668 * 10^{-2} eV/K
N -3.04101400 -1.89886100 0.01488800
N -2.23075700 0.37932000 -0.02017000
N -2.34433800 2.79599200 -0.05589800
N -0.04950400 2.03525100 -0.11626700
N 2.36833700 2.09608800 -0.18774800
N 1.56328600 -0.18261400 -0.13481300
N 1.66650200 -2.59995700 -0.11675400
N -0.62454400 -1.83642500 -0.03128900
C -3.19660800 -0.59684600 0.03764100
C -4.51891900 0.01649300 0.07494800
C -2.87304900 1.60384300 -0.00956600
C -4.31529500 1.39767400 0.04678400
C -5.79610000 -0.52855700 0.12311900
C -6.87027500 0.35798600 0.14074300
C -6.66598000 1.74495200 0.11291700

C -5.38301600 2.28570000 0.06620700
C -1.02822000 2.98807600 -0.11189200
C -0.42843900 4.29432100 -0.18134200
C 1.15676800 2.66486300 -0.19698700
C 0.96397000 4.08927600 -0.23654500
C 1.85051500 5.16811100 -0.30715500
C -0.96748500 5.58257800 -0.19855400
C 1.30950500 6.44359200 -0.32261700
C -0.08509200 6.64853000 -0.26926700
C 2.53276800 0.79002000 -0.14518900
C 3.85169800 0.18051300 -0.10695000
C 3.64256600 -1.20183800 -0.06681600
C 2.20360800 -1.40079600 -0.09715600
C 4.71267700 -2.09151800 -0.01443300
C 5.99771400 -1.55528700 -0.02045700
C 6.20556600 -0.16853900 -0.06935200
C 5.13405700 0.71843200 -0.10904700
C 0.34883600 -2.79483000 -0.10271100
C -1.83452700 -2.46474700 -0.02167200
C -0.25394800 -4.10154100 -0.13424000
C -1.64361800 -3.89171500 -0.08196200
C 0.27707700 -5.39105600 -0.19954800
C -2.53807400 -4.96279600 -0.09428600
C -0.61452100 -6.45237600 -0.21382600
C -2.00691800 -6.24139200 -0.16170100
H -5.93786300 -1.60426800 0.14903700
H -7.88451400 -0.02838200 0.17856100
H -7.52647600 2.40742600 0.12890700
H -5.21075700 3.35683900 0.04455600
H 2.92293200 5.00291100 -0.35044100
H -2.04207600 5.72843600 -0.15740500
H 1.96789600 7.30561500 -0.37852200
H -0.46925100 7.66427800 -0.28493100
H 6.85593300 -2.21943400 0.01927400
H 7.22096700 0.21578400 -0.07319900
H 5.28517800 1.79273000 -0.14498800
H 4.54279100 -3.16314100 0.03883000
H 1.35042900 -5.54811100 -0.23859300
H -3.60821500 -4.78753100 -0.04984600
H -0.23681500 -7.46932400 -0.26589100
H -2.67161900 -7.10011700 -0.17370700
Fe -0.34021000 0.09933100 -0.14151700
O -0.44786000 0.07320000 -2.24801500
H -1.23539500 -0.35138500 -2.64014200
H 0.36869900 -0.19489800 -2.76464000
O -0.14549600 0.12469800 2.17367800
H 0.50588100 0.82747400 2.35872900
H 0.35201200 -0.71766000 2.34268000
O 1.74032900 -0.47750700 -3.50854300
H 2.34816400 0.26633800 -3.34113700
H 2.21200900 -1.30783200 -3.30298800

O 1.88705400 2.03820900 2.75371700
H 1.52756700 2.89190100 3.01480400
H 2.36542400 2.20369400 1.92635800
O 1.27062900 -2.00088600 2.93541900
H 1.73107600 -2.53769400 2.28239200
H 1.94112300 -1.45319600 3.41864700
O -2.86695900 -1.06222700 -3.01657000
H -3.07543600 -1.71690100 -2.33837000
H -3.51055000 -0.36188600 -2.86638300
O 3.01443900 -2.85622700 -2.77006900
H 2.54585000 -3.10220100 -1.95712600
H 3.88707500 -2.59590200 -2.45665600
O 2.45599400 -0.20031700 4.45583900
H 2.49000600 0.63204200 3.96088600
H 1.57001100 -0.18728800 4.87367900
O 3.02639800 1.97035800 -3.07169100
H 2.30849700 2.49394200 -3.44179600
H 3.03035100 2.20524400 -2.13121600
O -0.25155700 -0.57913400 4.95155100
H -0.12804000 -1.50141300 4.69297300
H -0.57039900 -0.17834000 4.12639500

$FePc(H_2O)_{10}^+$:
ZPE = 7.488 eV
S = 1.742 * 10^{-2} eV/K
N -3.26242600 -1.51307400 -0.08036100
N -2.20994700 0.66113000 -0.09739700
N -1.99858800 3.06764200 -0.13744800
N 0.18003800 2.02115300 -0.14383900
N 2.58479200 1.80438500 -0.17358700
N 1.53762600 -0.37033600 -0.08875000
N 1.31736500 -2.77735200 -0.05767600
N -0.85684200 -1.72856300 -0.04574400
C -3.27977100 -0.19250000 -0.07101200
C -4.51082300 0.57189600 -0.05281600
C -2.68947800 1.94885800 -0.10027000
C -4.13811000 1.92235000 -0.06858500
C -5.84723100 0.18715900 -0.01958100
C -6.79919600 1.20099800 -0.00677100
C -6.42546900 2.55508100 -0.02484200
C -5.08854700 2.93745600 -0.05513900
C -0.68052300 3.08920800 -0.16751100
C 0.08168700 4.31804100 -0.22973000
C 1.45959500 2.50187600 -0.19898700
C 1.43470300 3.94799900 -0.25103400
C 2.44588500 4.90489600 -0.30437900
C -0.30582000 5.65459300 -0.26496400
C 2.05905800 6.23930200 -0.33982400
C 0.70264100 6.60889500 -0.32097600
C 2.60853300 0.48061400 -0.10771600
C 3.83618000 -0.28074700 -0.04897500

C 3.45778500 -1.63019300 0.01129900
C 2.01359700 -1.65164000 -0.03366300
C 4.40932100 -2.64729100 0.08080500
C 5.74667400 -2.27174300 0.07499400
C 6.12531600 -0.91871000 0.00287500
C 5.17809800 0.09440500 -0.05784900
C -0.00195100 -2.80158200 -0.06973300
C -2.13967100 -2.20862400 -0.06292300
C -0.76598400 -4.03113500 -0.09249600
C -2.11641700 -3.65607100 -0.08617000
C -0.38641100 -5.36963300 -0.11172800
C -3.13385100 -4.60473400 -0.09855700
C -1.40204400 -6.31889300 -0.12760500
C -2.75564600 -5.94232600 -0.12077300
H -6.12581000 -0.86120300 0.00052700
H -7.85380800 0.94508400 0.02029600
H -7.19969900 3.31589100 -0.01276000
H -4.78954300 3.98006700 -0.06722700
H 3.49212500 4.61561700 -0.31685900
H -1.35541500 5.92812000 -0.24912500
H 2.81675100 7.01557600 -0.38316700
H 0.44288200 7.66243200 -0.35065500
H 6.51817600 -3.03342900 0.13040400
H 7.18093600 -0.66647800 -0.00464400
H 5.46665600 1.13820500 -0.12041900
H 4.11025200 -3.68902600 0.14830800
H 0.66035000 -5.65512100 -0.11345600
H -4.17607600 -4.30380300 -0.08687400
H -1.14809500 -7.37415300 -0.14389500
H -3.51810200 -6.71488400 -0.13073100
Fe -0.34078800 0.14587200 -0.16936400
O -0.37068100 0.11211400 -2.25390300
H -1.09505700 -0.42609200 -2.63825000
H 0.50439000 -0.12549900 -2.71910000
O -0.21519200 0.16651400 2.14238300
H 0.50188600 0.78876600 2.37272200
H 0.15487800 -0.73616000 2.36793600
O 1.84089200 -0.46542100 -3.34377400
H 2.57485800 0.16051200 -3.18849700
H 2.17596500 -1.37293300 -3.20057200
O 1.98982200 1.79415000 2.84349700
H 1.80926100 2.64048200 3.26595300
H 2.58671600 1.98648500 2.10823900
O 0.82283700 -2.07254100 2.99541000
H 1.27757900 -2.72807800 2.45900100
H 1.50115100 -1.62798000 3.57538700
O -2.44266100 -1.45910000 -3.08741500
H -3.18315200 -1.56560500 -2.48038100
H -2.82771600 -1.33199700 -3.95922600
O 2.67130200 -3.04510000 -2.78634300
H 2.22076400 -3.35302900 -1.98851800

H 3.60718300 -3.07471200 -2.56355700
O 2.16785600 -0.50280800 4.58933100
H 2.39127300 0.29286900 4.08542200
H 1.29375400 -0.29402600 4.98044400
O 3.77189000 1.46394800 -2.85031400
H 3.72333200 2.13741000 -3.53543200
H 3.55762700 1.91330100 -2.02024100
O -0.55233100 -0.26423800 4.98175600
H -0.71237400 -1.21420700 4.94865300
H -0.79017800 0.03306700 4.09015700

---

12 Explicit H_2O molecules:

$FePc(H_2O)_{12}$:
ZPE = 8.969 eV
S = 2.064 * 10^{-2} eV/K
N -2.92370600 -1.92156900 -0.25868500
N -2.10633300 0.34667800 -0.11881700
N -2.15179000 2.76560300 -0.04694400
N 0.13004600 1.95757600 -0.09593700
N 2.54478600 2.02216700 -0.03098400
N 1.73147900 -0.25241500 -0.07520700
N 1.76130900 -2.67304600 -0.14784900
N -0.51084600 -1.85065400 -0.19796900
C -3.07609600 -0.60838700 -0.14706700
C -4.38208100 0.01156600 -0.07905700
C -2.72472000 1.57756300 -0.04468800
C -4.15933500 1.39460600 -0.01193200
C -5.66855800 -0.52095200 -0.07405900
C -6.73023400 0.37151100 -0.00282700
C -6.50881600 1.75893100 0.06211000
C -5.22509400 2.28998800 0.05894200
C -0.84598600 2.93503300 -0.08143000
C -0.22142900 4.24024000 -0.08362400
C 1.34394800 2.58167400 -0.08916900
C 1.16404900 4.01950900 -0.08899300
C 2.07003400 5.07853900 -0.08097600
C -0.74001600 5.53350600 -0.07441300
C 1.55150400 6.36750400 -0.07428200
C 0.16294700 6.58952200 -0.07161900
C 2.70327300 0.69877600 -0.00943700
C 4.00086700 0.07416700 0.08454000
C 3.76539300 -1.31211500 0.08039900
C 2.33971400 -1.48074700 -0.03407300
C 4.82675800 -2.21932000 0.15066200
C 6.11323300 -1.70181700 0.21465200
C 6.34657100 -0.31300100 0.21415000
C 5.29525200 0.58969900 0.15176500
C 0.46093700 -2.83143700 -0.23741500

C -1.72799700 -2.47224200 -0.27888100
C -0.16445200 -4.13625600 -0.36393200
C -1.54390700 -3.90999000 -0.39179900
C 0.35446700 -5.42355600 -0.44482100
C -2.45213200 -4.95584500 -0.50028500
C -0.55174300 -6.47378800 -0.55851900
C -1.93590100 -6.24512900 -0.58610300
H -5.82078900 -1.59427800 -0.12229400
H -7.74935800 -0.00306300 0.00339500
H -7.36353500 2.42712500 0.11550700
H -5.04748000 3.35952500 0.10787700
H 3.14026700 4.89387600 -0.08065400
H -1.81276000 5.69710700 -0.07097100
H 2.22523000 7.21907400 -0.07157800
H -0.20776800 7.61042800 -0.06729700
H 6.96036600 -2.37923300 0.26984100
H 7.36777500 0.05216100 0.26221100
H 5.46587300 1.66165700 0.14417600
H 4.64149800 -3.28948600 0.16032800
H 1.42592800 -5.59451300 -0.41994300
H -3.51945200 -4.76085900 -0.51511600
H -0.18279700 -7.49300900 -0.62672800
H -2.61156500 -7.09043400 -0.67474200
Fe -0.18920000 0.05215000 -0.17376600
O -0.16919900 0.12920800 -2.34487000
H -0.80131900 -0.51040900 -2.74989600
H 0.73179600 0.04570500 -2.79199000
O -0.16111900 0.01437500 2.07719900
H 0.44186900 0.70015000 2.42180600
H 0.22768100 -0.85960400 2.36749600
O 2.15981600 -0.06244100 -3.41360600
H 2.78093800 0.64537400 -3.15559000
H 2.60070200 -0.92063000 -3.25881400
O 1.71949900 1.90336400 2.93421100
H 1.36076900 2.78663300 3.06786100
H 2.33359800 1.99217000 2.19037900
O 1.00744500 -2.15935900 2.96297300
H 1.58216800 -2.62318500 2.34538200
H 1.57306100 -1.62277600 3.58010800
O -2.37824200 -1.02964700 -3.32075200
H -2.92432000 -1.46707000 -2.65950800
H -2.67102400 -0.09468000 -3.33455400
O 3.30397100 -2.54162000 -2.82115600
H 2.74132300 -2.92055600 -2.13109000
H 4.12480600 -2.34690100 -2.35534100
O 1.85184500 -0.41220800 4.71617300
H 1.94241000 0.45410600 4.29634400
H 0.91535900 -0.46790400 5.01366400
O 3.65740000 2.18496100 -2.68860500
H 3.17382700 2.87022400 -3.16010300
H 3.45965900 2.34435100 -1.75020200

O -0.74226700 -1.14330700 5.08905300
H -0.54508600 -1.95910300 4.61289400
H -1.49308900 -0.76565400 4.58837200
O -2.58515000 -0.26001000 3.20928000
H -1.78921900 -0.02042900 2.69154700
H -3.11812100 0.53807100 3.25443400
O -2.28574300 1.70615900 -3.20346400
H -1.38792800 1.46065800 -2.91236600
H -2.64546300 2.22678900 -2.47860200

$FePc(H_2O)_{12}^+$:
ZPE = 8.985 eV
S = 2.063 * 10^{-2} eV/K
N 2.59922900 2.45218500 0.00234600
N 2.11241800 0.09594300 -0.20658800
N 2.49863700 -2.28033200 -0.39696800
N 0.12836000 -1.80304500 -0.37373400
N -2.25604000 -2.18506600 -0.36946200
N -1.76959100 0.15490600 -0.02519900
N -2.14617400 2.52502200 0.26380100
N 0.21854600 2.05368000 0.11820100
C 2.94095800 1.18468300 -0.12650500
C 4.32060800 0.75038700 -0.19817300
C 2.89541000 -1.02705700 -0.31563400
C 4.29180000 -0.64548400 -0.30857500
C 5.52061100 1.45245100 -0.16473500
C 6.69286900 0.70900700 -0.24953400
C 6.66389500 -0.69085800 -0.36197800
C 5.46224000 -1.39058700 -0.39299000
C 1.22375000 -2.62402000 -0.44429300
C 0.78719600 -3.99614000 -0.57985200
C -0.99690900 -2.58078900 -0.45104200
C -0.61597000 -3.96906300 -0.59351700
C -1.36006800 -5.14163900 -0.70826400
C 1.49131400 -5.19299500 -0.68846100
C -0.65745000 -6.33526500 -0.81627700
C 0.74880000 -6.36081000 -0.80727700
C -2.60078100 -0.92484400 -0.15683300
C -3.97608100 -0.49730200 -0.02729700
C -3.93727900 0.88606900 0.19897000
C -2.54327000 1.26666600 0.17055900
C -5.10614500 1.62599800 0.37482600
C -6.31197600 0.93997600 0.30309600
C -6.35069600 -0.44622500 0.06509000
C -5.18638600 -1.18402900 -0.09995200
C -0.87618000 2.87666800 0.23255800
C 1.34282900 2.83886700 0.11557600
C -0.43797000 4.25394100 0.31756700
C 0.96174300 4.22940900 0.24448600
C -1.13533900 5.45119900 0.44815000
C 1.71311800 5.39928900 0.29940000

C -0.38610800 6.62104400 0.49975500
C 1.01710400 6.59573700 0.42674500
H 5.53203700 2.53334700 -0.07338300
H 7.65198700 1.21688700 -0.22730900
H 7.60160800 -1.23412300 -0.42530200
H 5.43021500 -2.47121800 -0.48183200
H -2.44531200 -5.11853400 -0.70985000
H 2.57622700 -5.20094100 -0.68147900
H -1.20120200 -7.27028000 -0.90943900
H 1.25940100 -7.31471000 -0.89520600
H -7.24492400 1.47892100 0.43626000
H -7.31318400 -0.94496400 0.01058200
H -5.21346700 -2.25108600 -0.29201300
H -5.06650500 2.69322600 0.57079200
H -2.21860200 5.46379900 0.50726500
H 2.79621700 5.36807200 0.24720500
H -0.89170900 7.57645400 0.59948600
H 1.56459900 7.53189400 0.47264400
Fe 0.17273200 0.13220500 -0.16377500
O 0.07184300 0.41546100 -2.31552600
H 0.53899100 1.24095000 -2.60120800
H -0.86520900 0.38962100 -2.72821300
O 0.25949800 -0.27670800 2.02481700
H -0.48025800 -0.89203000 2.25277100
H 0.18021100 0.50188400 2.66444700
O -2.25449800 0.36093800 -3.28116400
H -2.80085200 -0.44289700 -3.17449600
H -2.80064100 1.13988900 -3.05223300
O -1.60353900 -2.07597000 2.75584800
H -0.99270700 -2.68489200 3.23154100
H -2.10507800 -2.58698800 2.11312300
O -0.00406400 1.45492300 3.88138700
H -0.65385900 2.16178600 3.84074200
H -0.36703900 0.77617400 4.50790400
O 1.87863000 2.18310500 -3.11604000
H 2.38231100 2.72579300 -2.50188300
H 2.41419600 1.37815600 -3.26669500
O -3.64749800 2.60298400 -2.47530200
H -3.23563600 2.98360900 -1.68882900
H -4.54223700 2.38402500 -2.19541900
O -1.01642000 -0.64846200 5.15062800
H -1.71005800 -0.95993300 4.55397800
H -0.38028700 -1.38512700 5.12866800
O -3.63276800 -2.00893200 -2.92263700
H -3.48520500 -2.62080400 -3.64971200
H -3.26431100 -2.43294700 -2.13287900
O 0.35179500 -2.96351200 4.27999300
H 0.56311400 -3.73334600 4.81421400
H 1.17053600 -2.71179400 3.77145600
O 2.26208000 -1.97683200 2.78930000
H 1.69562600 -1.25340100 2.44700700

H 2.58993500 -2.44518600 2.01483700
O 2.50802000 -0.46275900 -3.20728200
H 1.54892500 -0.47823900 -3.02915000
H 2.63497300 -0.92103200 -4.04272300
