# Supplementary material for: Role of Water in Modulating the Fe3+/Fe2+ Redox Couple in Iron-Based Complexes and Single-Atom Catalysts
Source: J Phys Chem Lett. 2025 Sep 18;16(39):10049–57. doi: 10.1021/acs.jpclett.5c02424 (PMC12498497; doi:10.1021/acs.jpclett.5c02424)
Supplement: Supplementary file 2 [file jz5c02424_si_002.pdf]

jz-2025-02424z.R1

Name: Peer Review Information for "Role of Water in Modulating the Fe^{3+}/Fe^{2+} Redox Couple in Iron-Based Complexes and Single-Atom Catalysts"

First Round of Reviewer Comments

Reviewer: 1

Comments to the Author

I will recommend the paper by Bonardi et al for publication in JPCL if the authors make a major revision.

First of all, the list of citations is long, but I think the authors missed the fact that capturing adsorbate-solvent interactions by means of micro-solvation has been done for other materials and better agreement was also found with experiments (Applied Catalysis B: Environmental 276, 2020, 119147; Catalysis Today 323, 2019, 35-43; Catalysis Today 312, 2018, 118-125). For phthalocyanines the role of ligands, extra electrons, the actual state of the metal site, etc. under reaction conditions has been inspected for CO2 reduction (Nat Commun 14, 3401 (2023); J. Mater. Chem. A, 2024, 12, 31547-31556). The authors here apply this to SACs and Fe redox couples, which is new and interesting, but still credit should be given to previous works.

Second, the authors neglected a solvation term in the formula of page 9. It might be dangerous to discard these two terms on the basis of two old papers. Can the authors make their own test?

Third and most importantly, I think that the authors do not know if water is electrochemically stable at the potentials they consider. In other words, the authors should consider the fact that *OH might be formed on the SACs. A potential of 0.6-0.8 V vs SHE seems enough to form *OH on the Fe site, which would also oxidize the site from Fe(II) to Fe(III). Hence, the authors should calculate the *OH adsorption potential from water on the Fe site using the CHE (*+H2O->*OH+H++e-). In doing so, solvation should be incorporated. This potential will tell them if water is truly adsorbed at 0.6-0.8 V vs SHE or if *OH is at the site instead.

Minor details: the keywords are all acronyms, please spell them out. Oxidation states using roman numbers are generally positive, so no need to say +II, just II suffices.

Reviewer: 2

Comments to the Author

The paper by Bonardi et al. reports a relevant aspect related to the evaluation of the Fe^{2+}/Fe^{3+} redox potentials and highlights how a correct determination is only possible when considering the ligand effect of one or two coordinated water molecules. The study is scientifically sound, interesting, and well-suited for this journal; therefore, I recommend it for publication.

However, a few points of curiosity arise:

The coordination of water is hypothesized to be axial, but when the complex is supported on an electrode surface or, more specifically, when the active site is embedded within a graphenic/graphitic plane, the square-pyramidal geometry seems to be the only possible configuration.

To what extent could the support or the chemical environment influence both the standard potential of the Fe^{2+}/Fe^{3+} redox couple and the coordination of water molecules?

Could a water molecule potentially coordinate by intercalating between the metal center and the support? Are there any experimental or theoretical evidences for this possibility, or would the system be completely unstable?

If the system were in solution in the presence of ligands stronger than water—for instance, nitrite (NO_2^-), traces of oxygen, or other non-innocent anions originating from the electrolyte—would it be reasonable to expect an effect similar to that observed for water coordination?

Moreover, is such an effect scalable as a function of the ligand strength, or would the behavior fundamentally differ?

Author's Response to Peer Review Comments:

Milano, September 10th, 2025

Senior Editor of

The Journal of Physical Chemistry Letters

Dear Editor,

Thanks for your consideration of our manuscript jz-2025-02424z entitled "Role of Water in Modulating the Fe^{3+}/Fe^{2+} Redox Couple in Iron-Based Complexes and Single-Atom Catalysts".

We are now submitting a revised version. We would like to thank you for sending our manuscript to two experts who raised a series of suggestions that triggered a series of improvements of our work. All reviewers recognized the novelty and impact of our work, giving positive comments and marks. The first reviewer was very positive in recommending publication after addressing a series of revisions. The second reviewer recommend published as is, and raised a series of suggestions. We have carefully revised the manuscript and addressed the useful suggestions provided by the reviewers.

We are confident that with the improvements triggered by the reviewers our work better fulfils the high-quality standards of The Journal of Physical Chemistry Letters.

Cordially yours,

Giovanni Di Liberto and Gianfranco Pacchioni

Changes to the text are highlighted in yellow

Reviewer 1

The reviewer wrote: "*I think the authors missed the fact that capturing adsorbate-solvent interactions by means of micro-solvation has been done for other materials and better agreement was also found with experiments (Applied Catalysis B: Environmental 276, 2020, 119147; Catalysis Today 323, 2019, 35-43; Catalysis Today 312, 2018, 118-125). For phthalocyanines the role of ligands, extra electrons, the actual state of the metal site, etc. under reaction conditions has been inspected for CO2 reduction (Nat Commun 14, 3401 (2023); J. Mater. Chem. A, 2024, 12, 31547-31556).*"

Our reply: We apologize for the incorrect reference to the microsolvation scheme and have revised the main text accordingly, following the suggestion. This better justifies our choice of investigating solvent effect with clusters of increasing size.

"In order to corroborate this, we have modelled an FePc complex with explicit water molecules, as microsolvation not only offers a practical way to account for explicit solvent effects with acceptable computational costs, it also provided better agreement with experiments than implicit solvation schemes[73–75]."

"Consequently, accurate quantum chemical modeling of FePc chemistry must explicitly account for the presence of water, a conclusion that holds true also for Fe@N-Gr and Fe@CN SACs as well and highlights the crucial role of ligands in defining the metal center state, as already shown in previous studies which investigated key aspects in other metal phtalocyanines single-site catalysts[76-77]."

The reviewer wrote: "*Second, the authors neglected a solvation term in the formula of page 9. It might be dangerous to discard these two terms on the basis of two old papers. Can the authors make their own test?*"

Our reply: We really appreciate this suggestion. We reconsidered the discussion of the main equation to calculate the oxidation potential. In our calculations, the DFT energy of the process involves the optimization of the phthalocyanines systems with iron having oxidation state either II or III. At the same time, the water environment is allowed to relax in both cases as a function of the charge of the metal. Also, zero-point energy and entropic contributions are explicitly accounted

for. Therefore, the reorganization energy of the solvent is already included in our calculations. The missing term in the calculation is the full treatment of bulk water, which is the reason why we performed a systematic assessment of the results as a function of cluster size of water.

"*We estimated the oxidation potential of the Fe^{2+}/Fe^{3+} pair against the Standard Hydrogen Electrode (SHE). We first calculated the free energy to remove one electron from the system with respect to the vacuum level, by including entropic and zero-point energy contributions and correcting the value with the absolute value of the SHE against the vacuum level. This term is calculated by fully relaxing the atomic coordinates of the systems and of the water clusters. Therefore, it explicitly considers the energetic term related to the change in oxidation state of the metal atoms and to some extent the reorganization energy of the water clusters.*

$$V_{Fe2+/Fe3+} = \Delta E_{DFT} - T\Delta S + \Delta E_{ZPE} =$$

$$= (E_{[FePc]^+} - E_{[FePc]}) - T(S_{[FePc]^+} - S_{[FePc]}) + (ZPE_{[FePc]^+} - ZPE_{[FePc]})$$

$E_{[FePc]}$ and $E_{[FePc]+}$ are the DFT energies of the FePc systems, $TS_{[FePc]}$ and $TS_{[FePc]+}$ are the entropic corrections and $ZPE_{[FePc]}$ and $ZPE_{[FePc]+}$ are the corresponding zero-point energies of the complexes. Finally, the main approximation of the calculation is the neglection of the contribution of bulk water, which is the main reason why we performed a systematic assessment of the calculated potential on the size of the water clusters."

The reviewer wrote: "*I think that the authors do not know if water is electrochemically stable at the potentials they consider. In other words, the authors should consider the fact that *OH might be formed on the SACs. A potential of 0.6-0.8 V vs SHE seems enough to form *OH on the Fe site, which would also oxidize the site from Fe(II) to Fe(III). Hence, the authors should calculate the *OH adsorption potential from water on the Fe site using the CHE (*+H2O->*OH+H++e-). In doing so, solvation should be incorporated. This potential will tell them if water is truly adsorbed at 0.6-0.8 V vs SHE or if *OH is at the site instead.*"

Our reply: The reviewer is right. We considered the formation of *OH as suggested by the reviewer. Interestingly, at the oxidation potential of FePc the formation of *OH is endergonic, although it depends on the nature of the cluster. More specifically, if we consider the formation of

*OH from one adsorbed water molecule, the □*G* becomes 0.28 eV at *E* = 0.8 V. If we start from the more plausible model with two coordinated water molecules, □*G* is equal to 0.78 eV at *E* = 0.8 V; this reduces to 0.35 eV only at the equilibrium potential of OER, *E* = 1.23 V.

"*An important aspect to assess is the formation of *OH species resulting from the oxidation of adsorbed water at the oxidation potential of FePc. We calculated the formation energy of *OH starting from two models having one and two adsorbed water molecules, respectively, on [Fe(III)Pc]$^+$; this corresponds to a square pyramidal and an octahedral-like coordination, respectively. The formation of *OH is endergonic at E = 0.80 V, although the value depends on the specific model adopted. The calculated Gibbs free energies are 0.28 eV and 0.75 eV for [Fe(III)Pc(H$_2$O)]$^+$ and [Fe(III)Pc(H$_2$O)$_2$]$^+$, respectively. It is interesting to observe that, if we consider the more likely [Fe(III)Pc(H$_2$O)$_2$]$^+$ catalyst model, the formation Gibbs free energy of [Fe(III)Pc(H$_2$O)(OH)]$^+$ is 0.35 eV at the equilibrium potential of OER, E = 1.23 V, which is compatible with excellent OER activity of the iron phthalocyanine-based systems, reporting overpotentials in the range 0.3-0.4 V at the reference value of current density 10 mA/cm^2.*[110]"

The reviewer wrote: "*The keywords are all acronyms, please spell them out. Oxidation states using roman numbers are generally positive, so no need to say +II, just II suffices.*" **Our reply**: We revised the text accordingly.

"*Keywords: Single Atom Catalysts, Density Functional Theory, Oxygen Evolution Reaction*"

Reviewer 2

The reviewer wrote: "*The coordination of water is hypothesized to be axial, but when the complex is supported on an electrode surface or, more specifically, when the active site is embedded within a graphenic/graphitic plane, the square-pyramidal geometry seems to be the only possible configuration.*

To what extent could the support or the chemical environment influence both the standard potential of the Fe^{2+}/Fe^{3+} redox couple and the coordination of water molecules?"

Our reply: This point is well taken. Indeed, the support plays a role in determining the catalytic activity. We better pointed out this aspect. Regarding the local coordination, one needs to assume a permeation degree of the solvent to have an octahedral-like coordination, otherwise only one water ligand is possible. Although we cannot provide an undisputed answer to this point, we added a discussion to underline its importance.

"The local coordination is then largely governed by the solvent, but it is also influenced by the nature of the support, as if solvent permeation cannot be assumed, a square-pyramidal geometry would be the only feasible one and the role of the support itself should be explicitly taken into account. In this context, the possible intercalation of water molecules between the metal center and the support would also deserve further investigation."

The reviewer wrote: "*Could a water molecule potentially coordinate by intercalating between the metal center and the support? Are there any experimental or theoretical evidences for this possibility, or would the system be completely unstable?*"

Our reply: This is point is related to the previous point. We cannot exclude that the solvent can penetrate through the support, but this would require a dedicated study once the support is known. We revised the text to underline the point (see above).

The reviewer wrote: "*If the system were in solution in the presence of ligands stronger than water— for instance, nitrite (NO_2^-), traces of oxygen, or other non-innocent anions originating from the electrolyte—would it be reasonable to expect an effect similar to that observed for water coordination?*
Moreover, is such an effect scalable as a function of the ligand strength, or would the behavior fundamentally differ?"

Our reply: Also this point is well taken. It raises two fundamental questions that we are planning to further investigate. The first one is the generalization of the message, assuming that other species can coordinate to the system. The second point raises the question of whether there is a straightforward correlation between adsorption strength and the nature of the ligand. This aspect would be particularly interesting, as we expect it to depend on the type of interaction, whether electrostatic or covalent [10.1038/s41557-023-01424-6].

"Further work will be focused on addressing two relevant questions: first, if other species coming from the reaction environment can effectively compete with H_2O; second, if there is a relationship between the adsorption strength and the nature of the ligand, likely dependent on the type of interaction. In this respect, we highlight a seminal work by Schumann et al. where it was reported that adsorbate binding on single-atom alloys is strongest when the dopant and adsorbate contribute a total of ten bonding electrons, but this rule holds mainly for covalent interactions, while electrostatic cases depend more on dopant charge.[111] *Investigation of these aspects is crucial as previous studies have shown that different electrolytes solutions lead to different behaviour with the redox process being sensitive to the presence of electrolyte anions, as some can poison the catalyst at modest concentrations.*[71] *While a dedicated investigation is needed to fully address these points, these considerations highlight the importance of both ligand identity and the local chemical environment in governing redox and catalytic properties."*

jz-2025-02424z.R2

Name: Peer Review Information for "Role of Water in Modulating the Fe^{3+}/Fe^{2+} Redox Couple in Iron-Based Complexes and Single-Atom Catalysts"

Second Round of Reviewer Comments

Reviewer: 1

Comments to the Author

I thank the authors for addressing my comments. I am satisfied with the revision and recommend their paper for publication in JPCL.

Author's Response to Peer Review Comments:

Milano, September 15th, 2025

Senior Editor of

The Journal of Physical Chemistry Letters

Dear Editor,

Thanks for your consideration of our manuscript jz-2025-02424z_R1 entitled "Role of Water in Modulating the Fe^{3+}/Fe^{2+} Redox Couple in Iron-Based Complexes and Single-Atom Catalysts".

We are glad that the manuscript was considered suitable for publication. We are now submitting a revised version addressing the editorial requests. We also fixed a few typos in the text. No scientific changes have been made to the main text and ESI.

Cordially yours,

Giovanni Di Liberto and Gianfranco Pacchioni
